# A stimuli-responsive pillar[5]arene-based hybrid material with enhanced tunable multicolor luminescence and ion-sensing ability

**DOI:** 10.1093/nsr/nwaa281

**Published:** 2020-11-15

**Authors:** Xin-Yue Lou, Nan Song, Ying-Wei Yang

**Affiliations:** State Key Laboratory of Inorganic Synthesis and Preparative Chemistry, International Joint Research Laboratory of Nano-Micro Architecture Chemistry (NMAC), College of Chemistry, Jilin University, Changchun 130012, China; State Key Laboratory of Inorganic Synthesis and Preparative Chemistry, International Joint Research Laboratory of Nano-Micro Architecture Chemistry (NMAC), College of Chemistry, Jilin University, Changchun 130012, China; State Key Laboratory of Inorganic Synthesis and Preparative Chemistry, International Joint Research Laboratory of Nano-Micro Architecture Chemistry (NMAC), College of Chemistry, Jilin University, Changchun 130012, China

**Keywords:** aggregation-induced emission, supramolecular chemistry, luminescent materials, pillararenes, macrocycles

## Abstract

Tunable luminescent materials are becoming more and more important owing to their broad application potential in various fields. Here we construct a pillar[5]arene-based hybrid material with stimuli-responsive luminescent properties and ion-sensing abilities from a pyridine-modified conjugated pillar[5]arene and a planar chromophore oligo(phenylenevinylene) upon coordination of Cd (II) metal cores. This new material not only shows an optimized luminescence due to the minimized π–π stacking and efficient charge transfer properties benefitting from the existence of pillar[5]arene rings, but also exhibits tunable multicolor emission induced by different external stimuli including solvent, ions and acid, indicating great application potential as a fluorescent sensory material, especially for Fe^3+^. With this pillar[5]arene-based dual-ligand hybrid material, valid optimization and regulation on the fluorescence of the original chromophore have been achieved, which demonstrates a plausible strategy for the design of tunable solid-state luminescent materials and also a prototypical model for the effective regulation of fluorescent properties of planar π systems using synthetic macrocycle-based building blocks.

## Teaser

For higher tunability, brighter emission, and extra colors of luminescent materials, one can simply use some pillars (pillar[n]arenes) to facilitate material construction.

## INTRODUCTION

Tunable luminescent materials, especially organic photoluminescent materials with stimuli-responsive emission properties, have been highlighted as an exciting research topic in the past few decades owing to their prominent potential with regard to light-emitting diodes, [1] optoelectronic devices, [2] fluorescent sensing, [3] *in vivo* imaging, [4] anti-counterfeiting, [5] data storage [6] and information encryption [7]. However, applications of tunable fluorescent materials in solid states have been largely hampered because these luminescent systems generally require time-consuming organic synthesis procedures and suffer from reduced photoluminescence (PL) and poor dispersity in solution phases. Remarkably, the aggregation-induced emission (AIE) phenomenon has been widely studied since it was first brought up by Tang and coworkers in 2001 [8], and has been considered an important solution against the well-known self-quenching problems universally existing in luminescent materials such as dyes [9–12]. Stemming from the identical pursuit of enhanced luminescence, supramolecular approaches have been proposed for the optimization and manipulation of the fluorescent properties of dyes, and supramolecular materials that display adjustable luminescent features have already been developed in recent years [13–15]. Nevertheless, solid-state supramolecular materials with controllable luminescent outputs may bring new insights into the field of smart luminescent materials [5,16].

Pillar[n]arenes (pillarenes) [17,18], as a new type of supramolecular macrocycle, possess regulated symmetric pillar-shaped skeletons and π-electron rich hydrophobic cavities, which have been under intense investigation since first reported by Ogoshi and coworkers in 2008 [19]. Particularly, supramolecular fluorescent systems, including fluorescent polymeric materials [20,21] and supramolecular assemblies [22–24], have been fabricated using pillarenes as seminal building blocks, thanks to the versatile functionalization and unique host-guest properties. However, most relative research has been focused on the tuning of luminescence by taking advantage of the dynamic and reversible host-guest interactions of pillarenes, but the strategy of adjusting fluorescent properties directly by equipping chromophores with pillarene rings remains barely touched. For example, in 2019 we demonstrated a supramolecular approach of yielding intense fluorescence both in solution and solid states by covalently linking pillar[5]arene to the terminals of an anthracene derivative, and realized effective color tuning via host-guest interaction of pillar[5]arene and fluorescent guest molecules [25]. Apart from covalent modification, another powerful method for the integration of pillarenes and luminescent molecules is through coordination. Until today, several coordinated materials constructed with pillarene-based ligands have been developed, that is, pillarene-based metal-organic frameworks (MOFs) or pillarene coordination polymers [26–29]. This research has allowed us to anticipate that, by integrating pillarenes and chromophores into one coordinated architecture, luminescent hybrid materials might be afforded with unexpected structural and photophysical properties, such as stimuli-responsive features [30].

**Scheme 1.**
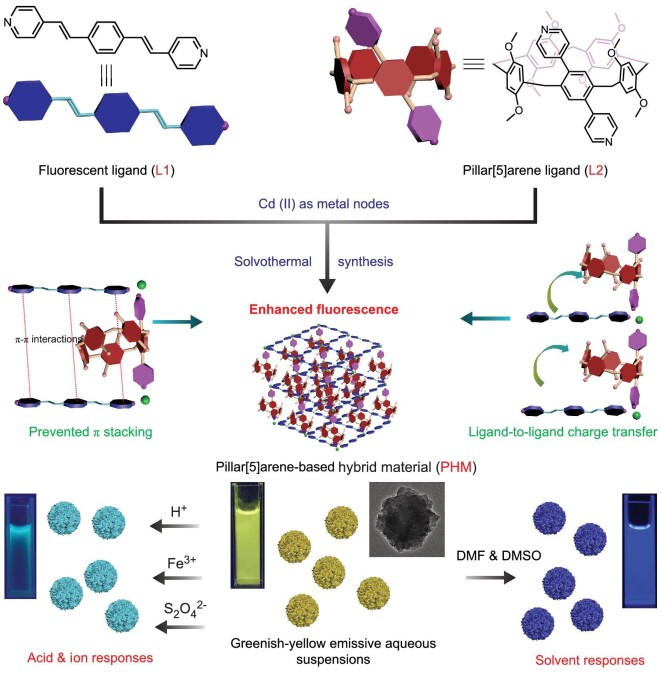
Schematic illustration of the synthesis of PHM, the proposed fluorescence tuning mechanisms, and the tunable luminescent responses of PHM toward different external stimuli.

Herein, we report on a dual-ligand pillar[5]arene-based hybrid material (PHM) with optimized fluorescence properties and stimuli-responsive luminescence (Scheme 1). A typical linear π-conjugated chromophore, oligo(phenylenevinylene) (OPV), was chosen as the fluorescent ligand (L1), which is known to possess adjustable electronic features depending on the molecular packing modes [31–33]. By immobilizing L1 and pyridine-modified conjugated pillar[5]arene (L2) to the Cd (II) metal cores via coordination, we successfully obtained an interesting luminescent material which possessed strong emission both in suspensions and solid states and displayed fascinating tunable multicolor luminescence in response to various stimulating factors, including solvents, ions and acid. In particular, the luminescent responses toward Fe^3+^ showed high selectivity and sensitivity, suggesting the great potential of this new material for application in fluorescent sensing [34,35]. The color-tuning responses toward various stimuli have been rationally attributed to the altered electronic distributions among the ligands through spectral analysis and theoretical calculations. By the construction of this tunable luminescent hybrid material, we provide a proof-of-concept model for the efficient adjustment of planar chromophores by synthetic macrocycles in the restricted space of coordination materials, and also enrich the family of tunable luminescent materials in solid states with stimuli-responsive properties.

## RESULTS AND DISCUSSION

### Preparation and characterization of PHM

**Figure 1.**
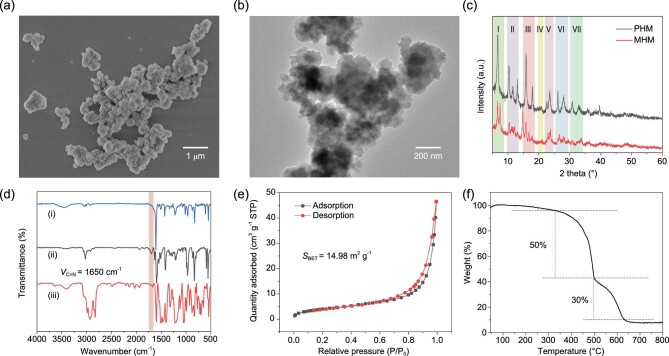
(a) SEM and (b) TEM images of PHM. (c) PXRD patterns of PHM (black) and MHM (red). (d) Fourier transform infrared (FTIR) spectra of (i) PHM, (ii) L1 and (iii) L2. (e) N_2_ adsorption and desorption, and (f) thermal gravimetric analysis (TGA) curves of PHM.

PHM was synthesized via a classical solvothermal protocol involving two organic ligands, i.e. OPV (L1) and pyridine-modified conjugated P5 (L2), in the presence of CdCl_2_•2.5H_2_O, which was yielded as a bright yellow powder. A counterpart monomer-based hybrid material (MHM) was also prepared following an identical synthetic route, except that L2 was replaced by bipyridine-containing monomer of L2 (M) (Supplementary Fig. S6). Scanning electron microscopy (SEM) and transmission electron microscopy (TEM) images indicated that PHM possesses a granular morphology at the micrometer scale and highly wrinkled surfaces with observable bulges and layers (Fig. 1a and b, and Supplementary Fig. S7). In contrast, the morphology of MHM was proven by SEM to be bulky and irregular without visible wrinkles or layers on the surfaces (Supplementary Fig. S8). Powder X-ray diffraction (PXRD) data suggested an ordered structure of PHM (Fig. 1c). In the comparison between the PXRD patterns of PHM and MHM, noticeable differences were found especially for the diffraction peaks in areas I and III, whereas overall similarities can be observed regarding the entire pattern, designating that even though structural differences exist between PHM and MHM, the two hybrid materials still adopt similar crystallinity. Nitrogen adsorption and desorption curves showed a type III nitrogen sorption isotherm, suggesting a mixture of mesopores and micropores of PHM with a surface area (*S*_BET_) of 14.98 m^2^ g^−1^ (Supplementary Figs S1e and S9).

Having acquired the morphological features and porosity parameters of PHM, further investigations were carried out to confirm the connection between ligands and metal nodes and to ratify the contents of each ligand. The coordination of the two ligands, namely L1 and L2, to the Cd (II) metal cores via pyridine units was confirmed by X-ray photoelectron spectroscopy (XPS) (Supplementary Fig. S10) and Fourier transform infrared (FTIR) spectroscopy (Fig. 1d). As expected, the vibrational peak of C=N (1650 cm^−1^) of pyridine groups in ligand molecules disappeared in the FTIR spectrum of PHM, attributing to the coordination of the nitrogen atoms to the Cd (II) cores [36]. Visual elemental mapping and energy dispersive spectroscopy (EDS) measurements were carried out to obtain information on the ligand content of PHM, whereby the ratio of L1 and L2 was calculated to be 2 : 1 according to the atomic percentage of N (5.82%) and O (7.54%) (Supplementary Figs S11 and S12, and Supplementary Table S1). The calculated ligand content was also consistent with the thermal gravimetric analysis (TGA) result (Fig. 1f), which revealed good thermal stability of PHM (up to 400°C) and also indicated a two-step weight loss covering 50% (400–500°C) and 30% (500–640°C), corresponding nicely to the L2 and L1 at the ratio of 1 : 2, respectively (Supplementary Table S2). The above analyses have provided confirmative evidence on the coordination between ligands and Cd (II) and have shed light on the ligand ratio of PHM. Based on these measurements, we came to the conclusion that L1 and L2 were coordinated to Cd (II) at a ratio of 2 : 1, and the predicted model of the PHM interior has been depicted in Scheme 1, whereby L2 entities locate in the axial opposite positions of the coordinated structure and L1 forms the planar part, representing the molecular arrangement with a minimized steric hindrance.

### Optical studies of PHM

**Figure 2.**
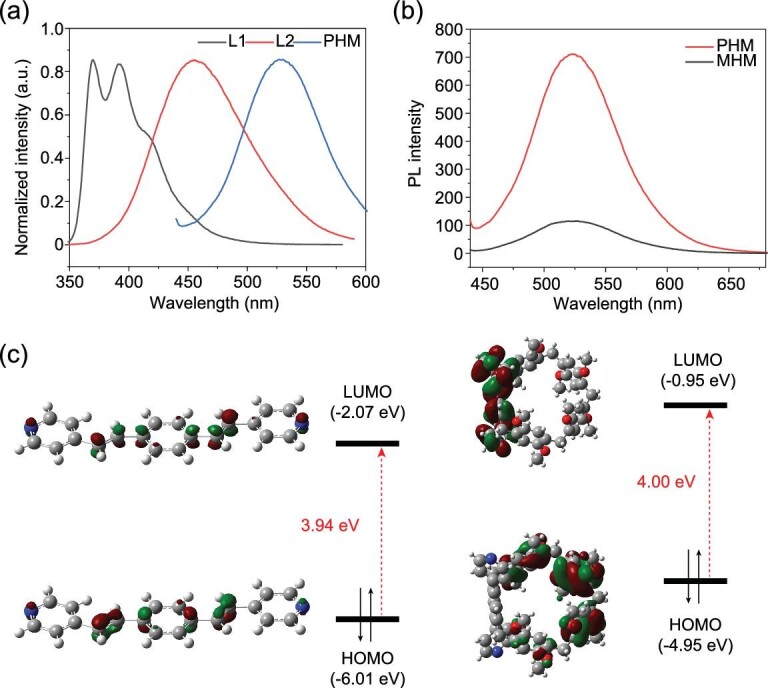
(a) Normalized PL spectra of L1 in CH_2_Cl_2_ (black), L2 in CH_2_Cl_2_ (red) and PHM in water suspension (blue). (b) PL spectra of PHM (red) and MHM (black) in water suspensions. (c) Molecular frontier orbitals of L1 (left) and L2 (right).

Considering the ordered packing modes of the chromophore and the rigid pillar[5]arene rings within the framework of PHM, the dual-ligand coordinated hybrid material was envisioned to give rise to remarkable fluorescent properties distinct from discrete OPV molecules. Different from the emission of 370–400 nm for L1 and 456 nm for L2, PHM exhibited a greenish-yellow fluorescence at 530 nm in both the solid state and water suspensions (Fig. 2a). From the literature [37,38], OPV dyes can produce highly tunable fluorescence that covers a broad emission spectrum, depending on the different molecular packing modes, such as J-aggregation. Based on this knowledge, the fluorescence at 530 nm can presumably be attributed to the intermolecular packing of L1 in the interior of PHM. This hypothesis was further confirmed by the solid-state fluorescence experiment of L1 (Supplementary Fig. S13) and the fluorescence spectrum of MHM whose emission maxima also located at 530 nm, indicating that the greenish-yellow fluorescence originated from L1 (Fig. 2b). Remarkably, PHM also exhibited rather high quantum efficiencies both in the powder state (14.61%) and water suspensions (28.05%) compared with those materials containing analogous emissive entities reported before (Supplementary Figs S14 and S15) [28,39,40].

However, although the two materials displayed identical emission peak wavelengths, PHM exhibited a 7-fold stronger fluorescence in intensity compared with MHM (Fig. 2b). Hypothetically, metal-to-ligand charge transfer (MLCT) may act as one facilitating factor in the fluorescence intensity, but considering that both PHM and MHM have been constructed via metal-ligand coordination and possess similar crystalline structures, simple MLCT is inadequate to explain this emission disparity. Hence, we deduce that the intense emission of PHM can be ascribed to the existence of another ligand, i.e. pillar[5]arene, which can potentially modulate the fluorescence properties of L1 from two perspectives. From the view of molecular packing, π–π interactions of L1 within the hybrid material can be efficiently avoided on account of the presence of the bulky rigid pillar[5]arene skeletons, largely minimizing the non-radiative relaxation of the OPV moieties and facilitating the radiative decay pathway. On the other hand, because of the adjacent location of L1 and L2 within PHM, changes in the electronic distributions should also be taken into consideration. A density functional theory (DFT) calculation was conducted (Supplementary Fig. S16, Supplementary Tables S3 and S4) [41], and referring to the calculated frontier molecular orbitals of the ligands (Fig. 2c), LUMO orbital of L1 occupies a lower energy level compared with L2, indicating that charge transfer (CT) processes are highly favorable between the conjugated pillar[5]arene and OPV chromophores, whereby the excited electrons of L2 can be transferred to the LUMO orbital of L1, thus efficiently intensifying the excited state of L1 and leading to the remarkable fluorescence enhancement. On the basis of the abovementioned two points, we deduce that the significant enhancement of the PL intensity of PHM undoubtedly originated from the favored CT between the two ligands, demonstrating the effective optimization of luminescent properties endowed by pillar[5]arene rings.

### Solvent responses of PHM

**Figure 3.**
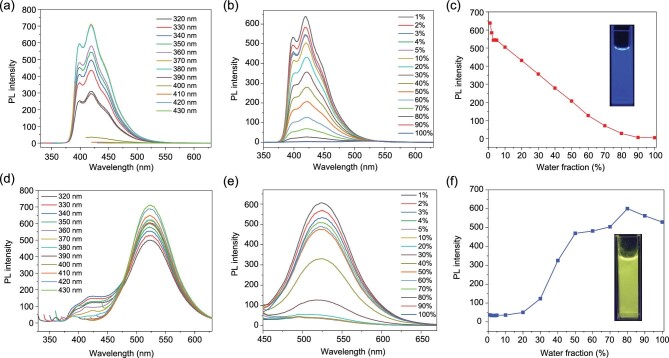
PL spectra of PHM in (a) DMSO and (d) H_2_O under the excitation wavelengths of 320–430 nm. PL spectra of PHM in DMSO suspension with increasing fraction of H_2_O excited at (b) 340 nm and (e) 430 nm. A plot of PL intensity of PHM suspensions at (c) 420 nm and (f) 530 nm vs. the fraction of water in the mixed solvent of DMSO–H_2_O.

Stimuli-responsive luminescent behaviors represent captivating features that are under much pursuit in the scope of fluorescent materials. Herein, we first investigated the luminescent properties of PHM in different solvents. An interesting phenomenon was observed that although the maximum emission peak of the material is located at 530 nm in solvents such as water, ethanol, acetone, and acetonitrile with the excitation wavelengths varying from 320 nm to 430 nm, a largely intensified emission at 400–450 nm can be observed when PHM was suspended in DMF or DMSO (Fig. 3 and Supplementary Fig. S17). By comparing the PL spectra of PHM in DMF or DMSO with the emission patterns of the ligands displayed in Fig. 2a, it can be deduced that the mazarine fluorescence of PHM is due to the monomer emission on account of the resemblance to the PL spectrum of discrete L1, except for a minor bathochromic shift (Supplementary Fig. S19).

Two feasible hypotheses have been proposed by us to explain the solvent-induced fluorescence switching, i.e. structural disruption of PHM, and blocked intermolecular interactions between L1 moieties. Since the solvothermal synthesis of PHM has been successfully conducted in DMF, the possibility of structural disorder caused by DMF can be excluded. Additionally, PHM powder that was once soaked in DMSO was washed and dried for PXRD measurement, and the result suggested the unchanged crystalline structure (Supplementary Fig. S20). Thus, these analysis results have eliminated the possibility of structural changes in the solvent-responsive behaviors of PHM, and on the contrary, have evidently pointed to the unfavored intermolecular interactions among L1 monomers caused by the two solvents, which consequently induced the monomer emission to triumph over the aggregated emission. The solvent responsiveness of PHM was further validated by the fluorescence experiments in DMSO—H_2_O mixed solvent with varied water fractions. When the water fraction increased, the fluorescence peak of 420 nm excited at 340 nm gradually weakened, and the emission at 530 nm exhibited a dramatic promotion under excitation of 430 nm, demonstrating a desirable luminescent response of PHM toward solvent constituents (Fig. 3c and f). Additionally, PHM also showed a higher quantum efficiency (39.51%) in DMSO, which is in good agreement with the intense fluorescence (Supplementary Fig. S21).

### Ion responses of PHM

**Figure 4.**
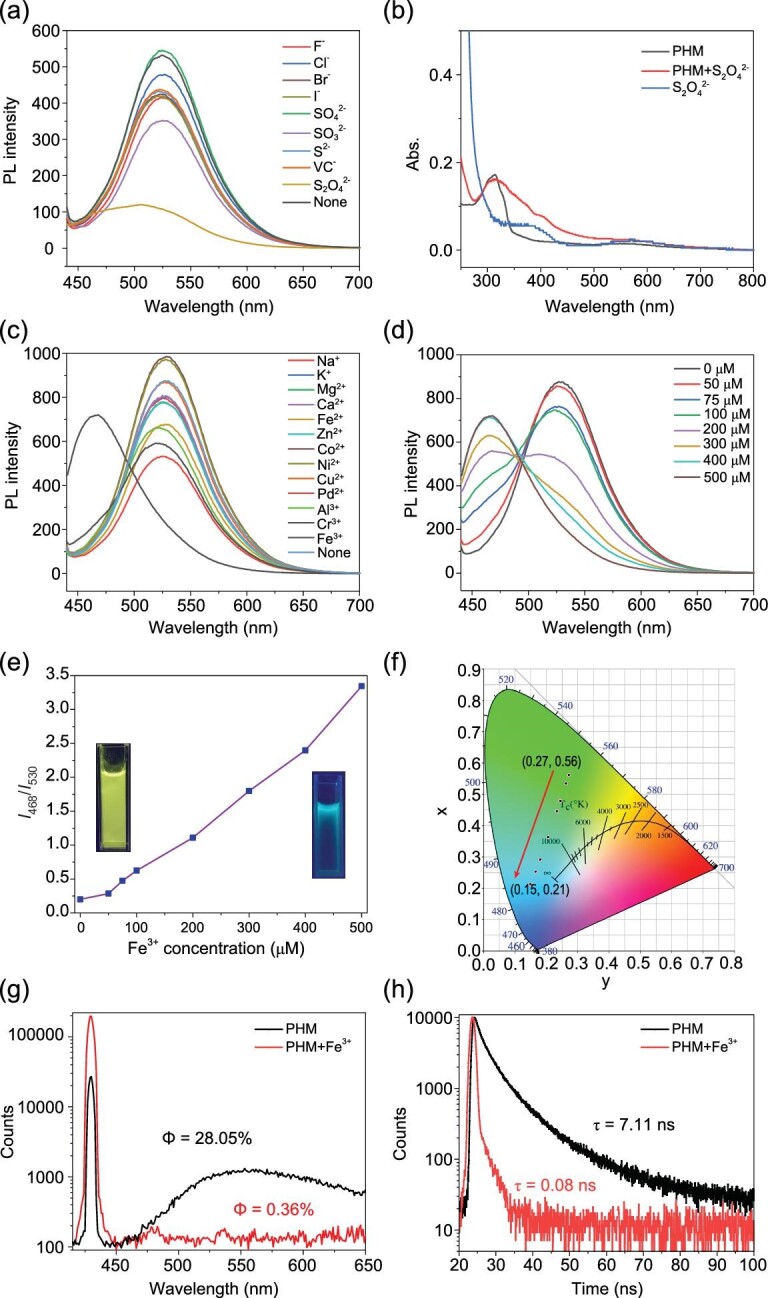
(a) Luminescent responses of PHM toward anion species. (b) UV-vis absorption spectra of PHM, PHM after treatment of S_2_O_4_^2−^ and S_2_O_4_^2−^. (c) Luminescent responses of PHM toward cation species. (d) PL spectra of PHM water suspension with different concentrations of Fe^3+^. (e) Plot of *I*_468_/*I*_530_ (ratio of intensity at 468 nm to intensity at 530 nm) versus Fe^3+^ concentration; inset: photographs before and after treatment of Fe^3+^ taken under a 365 nm UV lamp. (f) CIE coordinates in accordance with the spectra in (d). (g) Quantum yields and (h) fluorescence lifetimes of PHM before and after treatment of an excess of Fe^3+^. Note: λ_ex_ = 430 nm.

Another stimulus that can tune the emission color of PHM was found to be the addition of particular ion species. First, we investigated the luminescent responsiveness of PHM toward various anions, and discovered that S_2_O_4_^2−^, distinct from other anions, can induce fluorescence quenching of PHM suspension at 530 nm (Fig. 4a). UV-vis spectra of PHM treated with S_2_O_4_^2−^ also showed no changes in the absorption band (Fig. 4b), and the PXRD measurement displayed an identical diffraction pattern, suggesting that no electronic or structural changes occurred in the quenching response. In this response behavior, energy transfer from the chromophore to the anion could possibly be the main cause for the luminescence quenching.

Intriguingly, in the subsequent investigation of cation responsiveness, we observed that among all other cations, Fe^3+^ alone can induce the fluorescence quenching (at 530 nm) of PHM in aqueous suspensions, accompanied by the simultaneous appearance and intensification of a new emission peak at 468 nm (Fig. 4c). The gradual increase of Fe^3+^ concentration caused observable alteration of the fluorescence spectra of PHM, and an excess of ferric ions resulted in the thorough shift of the emission peak from 530 nm to 468 nm (Fig. 4d and e). Accordingly, the well-tuned emission color was also exhibited by a CIE chromaticity diagram, which shows that the PL of PHM has adjusted from greenish-yellow (0.27, 0.56) to cyan (0.15, 0.21) as Fe^3+^ increases, as shown in Fig. 4f, demonstrating the excellent color-tuning response toward ferric ions.

Based on the exclusive selectivity and efficient color tuning of PHM toward ferric ions given above, we expect that PHM could potentially serve as a fluorescent sensory material for Fe^3+^. Fluorescence titration was carried out by gradually adding Fe^3+^ aqueous solution into PHM aqueous suspension, whereby the increasing amount of Fe^3+^ led to a dramatic decrease of fluorescence intensity at 530 nm and slight emission enhancement at 468 nm. For the control experiment, titration with pure water was also conducted, in which the fluorescence of PHM only displayed a relatively trivial decrease due to dilution (Supplementary Fig. S22 and S23). Particularly, the quenching of PHM luminescence intensity at 530 nm displayed a very nice linearity as Fe^3+^ increased, and the limit of detection (LOD) of the titration was calculated to be 0.8 μM (Supplementary Fig. S21b), showing that PHM could not only perform selective responsiveness towards Fe^3+^ via obvious color changes, but also held promising potential in the application of Fe^3+^ sensing.

### Study of the color-tuning mechanism

The unique Fe^3+^ sensing capacity of PHM has inspired us to study the underlying mechanism of this color tuning process, and the collected fluorescence parameters have provided useful clues for exploration. First, we noticed that the emission pattern of PHM after treatment with Fe^3+^ almost totally overlapped with the fluorescence spectrum of L2 at 456 nm with a slight red shift of 12 nm (Supplementary Fig. S24). Additionally, PHM treated with Fe^3+^ possessed a dramatically reduced quantum yield and a much shortened excited-state lifetime compared with pristine PHM (Fig. 4g and h), which is also in good accordance with the poorly emissive nature of pillar[5]arene. As we have attributed the strong luminescence intensity of PHM to CT between the two ligands, all the above evidence has pointed to the possible interception of CT processes, which can straightforwardly lead to the weakened fluorescence of L1 and the promotion of L2 emission. Hence, the color-tuning effect of PHM emission by Fe^3+^ can be studied based on this plausible assumption.

**Figure 5.**
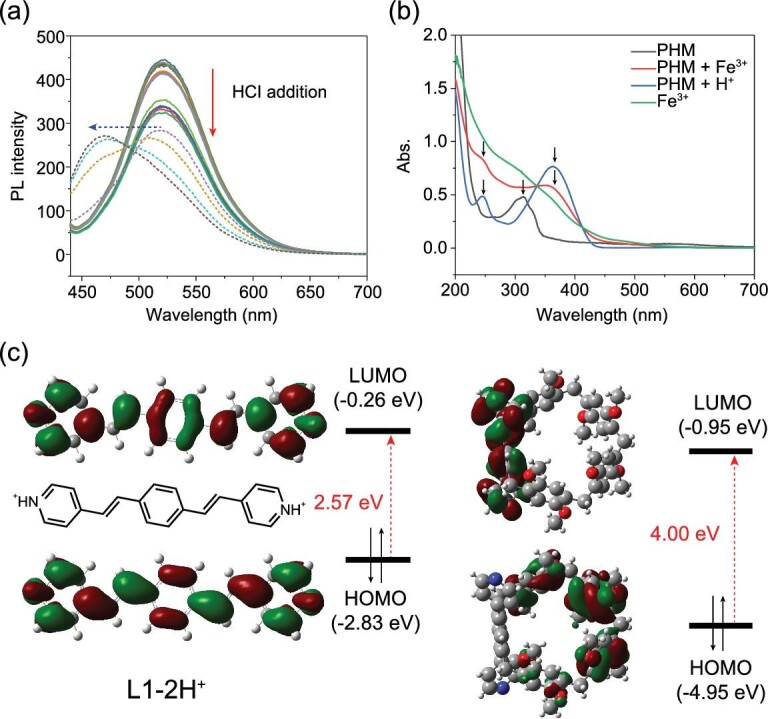
(a) PL spectra of PHM in water suspension upon increasing concentration of HCl (solid line: 0∼0.4 mM; dashed line: 0.4∼1 mM). (b) UV-vis absorption spectra of PHM (black), PHM + Fe^3+^ (red), PHM + H^+^ (blue) and Fe^3+^ (green). (c) Molecular frontier orbitals of L1–2H^+^ (left) and L2 (right).

Since Fe^3+^ possesses the strong ability of hydrolyzation in water and pyridine groups are vulnerable to acidic conditions [42,43], we reckoned that the blocked CT activities might be associated with the protonation of pyridine units of the ligands caused by Fe^3+^[42]. To testify to this, fluorescence spectra of PHM in water suspension treated by HCl were collected, and just as we expected, an excess of H^+^ caused the same peak-shifting and reduced quantum efficiency (Fig. 5a and Supplementary Fig. S27). Notably, the addition of base can lead to the recovery of the emission peak, back to 530 nm (Supplementary Fig. S28). From UV-vis absorption spectra of PHM, PHM treated by Fe^3+^ and by H^+^ (Fig. 5b), we also observed that, differently to the single absorption peak of the pristine material at 310 nm, PHM treated by Fe^3+^ or H^+^ displayed very similar absorption patterns with two new peaks at 245 nm and 364 nm, indicating that new species had been generated upon these two stimuli. The spectral analyses have powerfully revealed the seminal role of pyridine protonation and Fe^3+^ hydrolyzation in color-tuning responses.

After revealing that protonation of pyridine was the direct cause of the PL changes of PHM, DFT calculations were performed to further verify the hypothesis of prohibited CT processes. Because L1 possesses a more planar and extended π-conjugated system than L2, the pyridine groups of L1 would undergo protonation more easily. Thus, an extreme state of L1, in which both two pyridine units are protonated, has been taken as the model (L1–2H^+^), and its molecular frontier orbitals have been calculated (Fig. 5c). Both energy levels of HOMO and LUMO of L1–2H^+^ are higher than L1 itself, and the energy gap becomes smaller (2.57 eV). More importantly, the LUMO of L1–2H^+^ (−0.26 eV) is located at a higher energy level than that of L2 (−0.95 eV). Hence, the excited electrons of L2 would no longer be transferred to the LUMO of L1 simultaneously, resulting in a largely compromised CT process. By establishing this model, we have revealed that the raised LUMO of L1 after protonation is the main reason for the adjusted CT behaviors and the tuned fluorescence of PHM by Fe^3+^. In addition, PXRD measurements of PHM treated with Fe^3+^ and H^+^ were performed to confirm the intactness of the material structure (Supplementary Fig. S29), suggesting that no structural disturbance occurred, and that the changes in the electronic states had definitely acted as the dominant factor for the fluorescence response.

## CONCLUSION

In summary, a new type of highly tunable luminescent hybrid material, PHM, has been fabricated by integrating a fluorescent ligand and a pillar[5]arene-based ligand into one rigid coordinated architecture. The dual-ligand material displays superior fluorescent properties both in the powder phase and suspensions, originated from two synergetic factors involving the rigid pillar[5]arene skeleton and the favorable CT processes between ligands, and holds tremendous potential to address the challenges faced by traditional fluorescent materials without rigid macrocyclic entities. Moreover, PHM proves to be capable of generating multicolored luminescent responses toward different external stimuli including solvent contents, particular ions and the addition of acid. The luminescent responses have been confirmed to occur via tuning the electronic distributions among ligands without committing any structural disturbance of the material. The study suggests that the pillarene ring has contributed largely to the adjustment of fluorescent properties of PHM by acting as a rigid macrocyclic skeleton within the coordinated structure and a useful modulator in CT processes. Although this system is still far from perfect and certain issues such as the reversibility need to be further addressed, overall this study has paved a new way of adjusting and optimizing fluorescent features of planar dyes via integration with the rigid pillarene rings through coordination, providing a feasible approach for developing solid-state luminescent materials with tunable emission and desired stimuli-responsiveness.

## METHODS

### Preparation of PHM and MHM

CdCl_2_•2.5H_2_O was purchased from J&K Co. Ltd. (Beijing, China), and L1 and L2 were synthesized according to reported procedures. CdCl_2_•2.5H_2_O (22.8 mg, 0.1 mmol), L1 (24.8 mg, 0.1 mmol) and L2 (84.5 mg, 0.1 mmol) were dissolved in a mixture of DMF (8 mL) and isopropanol (2 mL) in a 25 mL Teflon tube. The mixture was stirred for 30 minutes. Then the solution was heated in the oven under high pressure at 120°C for 48 hours. The crude product was separated by centrifugation and washed several times with DMF, ethanol and water, respectively, to give a yellow insoluble powder as PHM. MHM was synthesized following the same procedure except that L2 was replaced by the pyridine-modified monomer M.

### Fluorescence experiments of PHM

All the fluorescent experiments were performed on a Shimadzu RF-5301PC spectrometer. The time-resolved fluorescence decay curves and quantum yields were measured on a FLS920 instrument (Edinburgh Instrument, UK). Quantum yields were calculated using an integrating sphere. The mother suspension of PHM was prepared by the simple dispersion of PHM in deionized water ([PHM] = 1 mg mL^−1^). All the optical experiments were conducted using diluted suspensions (100 times) of the mother suspension after sonication with a final concentration of 10 μg mL^−1^. MHM suspensions were prepared via an identical procedure. The fluorescence spectra were collected with the slit width set as (Ex: 5 nm, Em: 5 nm), except for PHM in the solvents of DMF and DMSO, whereby the slit width was (Ex: 3 nm, Em: 1.5 nm).

### Theoretical calculations and statistical analysis

DFT calculations were performed with the Gaussian 09 program (B3LYP, 3-21G*). The orbital representations were generated with Gaussview 5.0 (scaling radii of 75%, isovalue = 0.02).

Radiative decay rates and non-radiative decay rates have been calculated according to the equations as given below:

$$\begin{equation*}
\begin{array}{@{}l@{}} {{\rm{K}}_{\rm{r}}} = \Phi /\tau \\ {{\rm{K}}_{{\rm{nr}}}} = {\rm{ }}(1 - \Phi )/\tau \end{array}
\end{equation*}$$

## Supplementary Material

nwaa281_Supplemental_File
