# Supplementary material for: A stimuli-responsive pillar[5]arene-based hybrid material with enhanced tunable multicolor luminescence and ion-sensing ability
Source: Natl Sci Rev. 2020 Nov 15;8(6):nwaa281. doi: 10.1093/nsr/nwaa281 (PMC8288178; doi:10.1093/nsr/nwaa281)
Supplement: nwaa281_Supplemental_File [file nwaa281_supplemental_file.docx]

***Supplementary Data***

***for***

**A Stimuli-Responsive Pillar[5]arene-Based Hybrid Material with Enhanced Tunable Multicolor Luminescence and Ion Sensing Ability**

*Xin-Yue Lou, Nan Song and Ying-Wei Yang**

State Key Laboratory of Inorganic Synthesis and Preparative Chemistry,

International Joint Research Laboratory of Nano-Micro Architecture Chemistry (NMAC),

College of Chemistry, Jilin University, 2699 Qianjin Street, Changchun 130012, P. R. China

E-mail: [ywyang@jlu.edu.cn](mailto:ywyang@jlu.edu.cn)

**Table of Contents**

[1. Materials and syntheses of main compounds S2](#_Toc50209096)

[2. Preparation of the materials S9](#_Toc50209097)

[3. Structural analysis S10](#_Toc50209098)

[4. Fluorescence data of PHM S13](#_Toc50209099)

[5. DFT calculation S14](#_Toc50209100)

[6. Luminescent responses of PHM S18](#_Toc50209101)

[7. References S24](#_Toc50209102)

## 1. Materials and syntheses of main compounds

**Characterizations**

^1^H NMR spectra were recorded on a Bruker 400 MHz NMR spectrometer. UV-vis spectra were collected on a Shimadzu UV-2550 spectrometer. X-ray diffraction (XRD) measurements were carried out using a PANalytical B.V. Empyrean powder diffractometer. Fourier transform infrared (FTIR) spectra were recorded on a Vertex 80 V spectrometer. Scanning electron microscope (SEM) and energy dispersive spectroscopy (EDS) images were collected on a HITACHI SU8082 instrument. Thermogravimetric analysis (TGA) was performed under an air atmosphere with a heating rate of 10 ºC/min by using a NETZSCH STA499F3 QMS403D thermogravimetric analyzer. N_2_ sorption isotherms at 77 K were measured on a Quantachrome instruments ASiQMVH002-5 after pretreatment by heating the samples. The surface compositions of samples were obtained by X-ray photoelectron spectroscopy (XPS) measured on a PREVAC XPS/UPS System and the binding energies were calibrated with respect to the signal to the C 1s peak.

Starting materials and reagents such as terephthalaldehyde, 4-picoline, paraformaldehyde, 1,4-dimethoxybenzene, trifluoromethanesulfonic anhydride, pyridine-4-boronic acid, and 1,4-dibromobenzene were reagent grade and purchased from Aladdin Reagents (Shanghai, China) or Sigma-Aldrich (Darmstadt, Germany). The inorganic compounds were obtained from J&K Co. Ltd. (Beijing, China). All of the solvents in the study were reagent grade purchased from commercial sources and were used without further purification unless otherwise noted. The syntheses of the compounds L1, L2, and M were done according to slightly modified literature procedures.[S[1-S3](#_ENREF_1)]


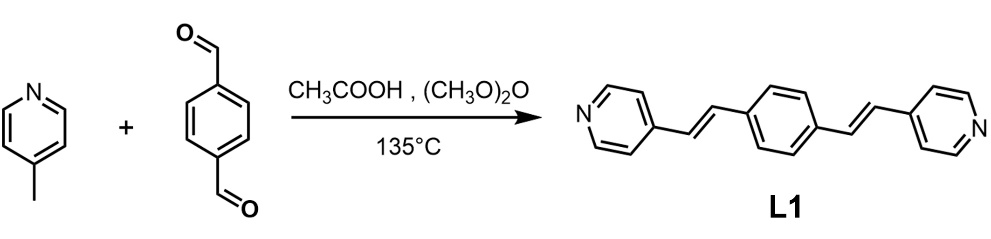


**Scheme S1.** Synthetic route of L1.

**Synthesis of L1:**

Terephthalaldehyde (3.4 g, 25.3 mmol) and 4-picoline (5 mL, 53.2 mL) were heated under reflux for 24 h in acetic acid (3cmL) and acetic anhydride (5 mL). The reaction mixture was cooled down to room temperature, and then placed in 100 mL of aqueous 6 M HCl. The resulting precipitate was collected and then washed repeatedly with deionized water. The filtrate was neutralized with 3 M NaOH. The crude product was separated from the mixture by filtration and purified by column chromatography (CH_2_Cl_2_: MeOH = 100:1) to afford **L1** as a yellow solid (yield: 60%). ^1^H NMR (400 MHz, CDCl_3_, 298 K): δ 8.60 (q, 4H), 7.58 (s, 4H), 7.40 (q, 4H), 7.35 (s, 1H), 7.30 (s, 1H), 7.10 (1H), 7.04 (s, 1H).

**
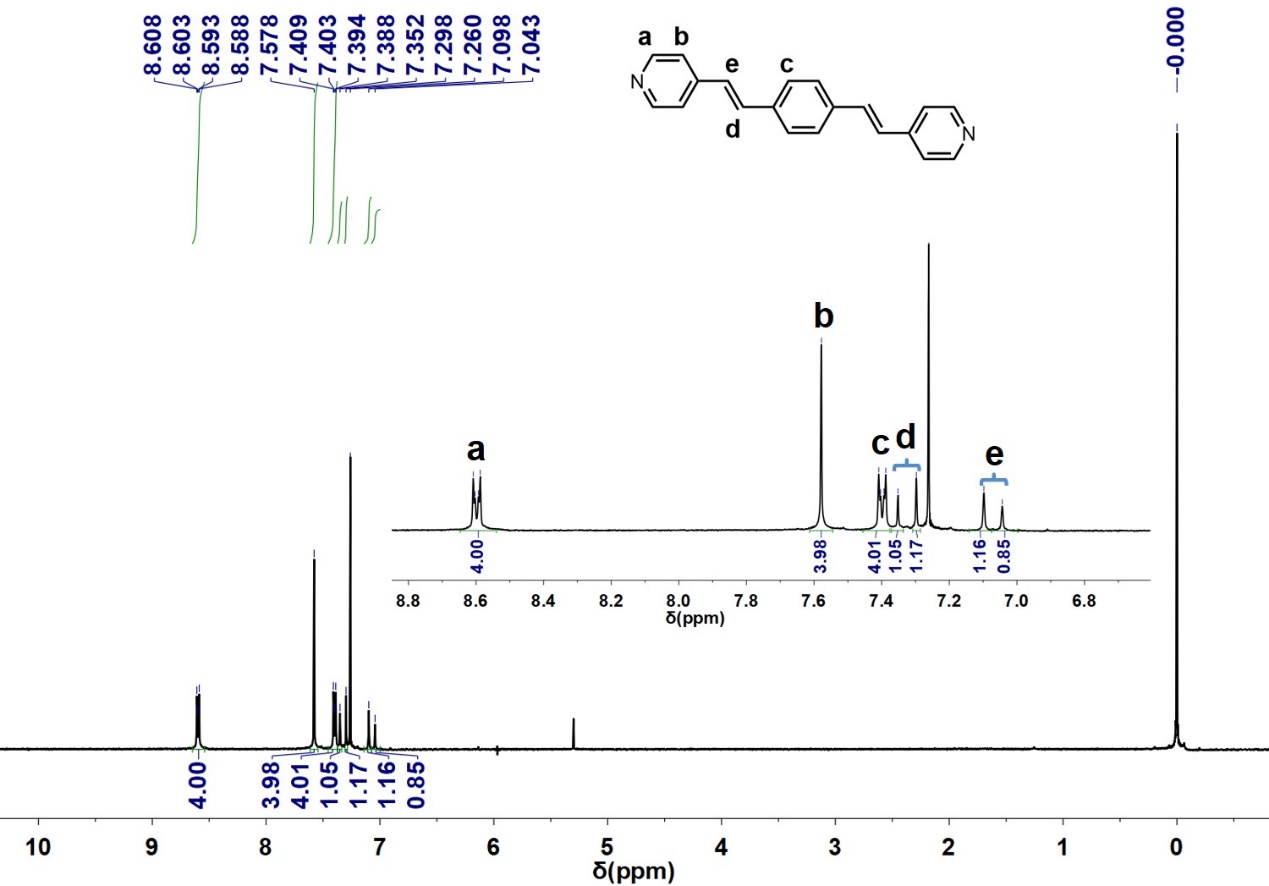
**

**Figure S1.** ^1^H NMR spectrum (400 MHz, CDCl_3_, 298 K) of **L1**.

**
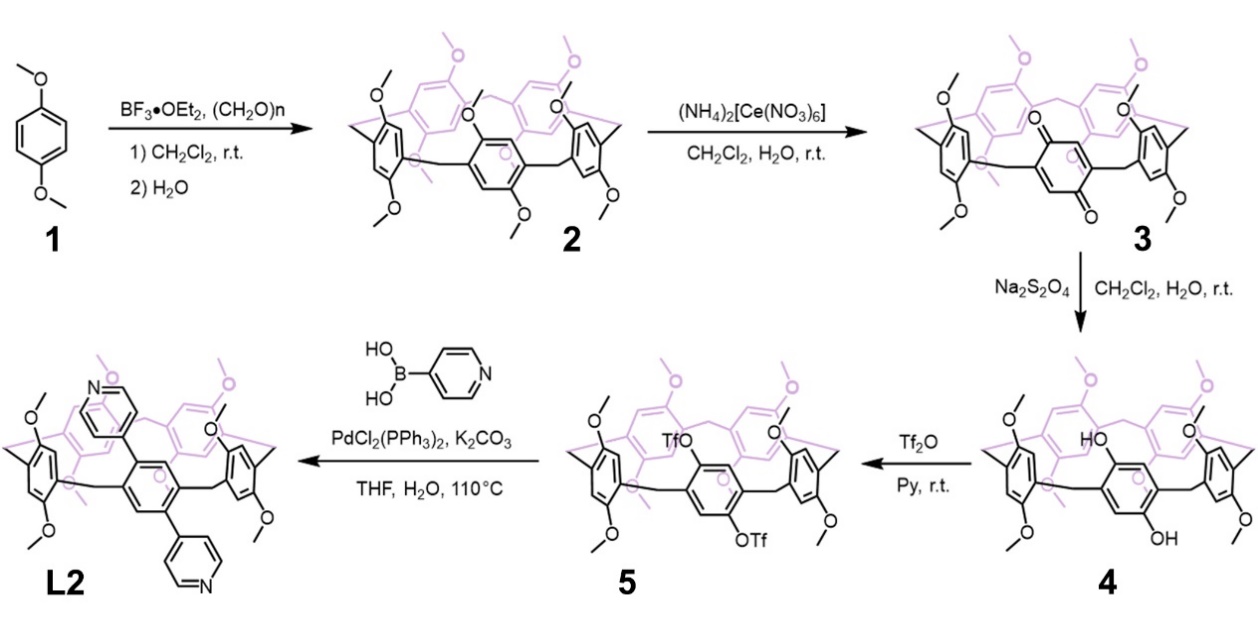
**

**Scheme S2.** Synthetic route of **L2**.

**Synthesis of Compound 2：**

Paraformaldehyde (0.450 g, 15 mmol) was added into a solution of 1, 4-dimethoxybenzene (7 g, 50 mmol) in dichloromethane (60 mL) under nitrogen protection. Then, boron trifluoride diethyl etherate (BF_3_·OEt_2_, 0.75 mL, 6 mmol) was added into the above solution and kept stirring at 0 °C for 130 min. After that, an aqueous solution of NaHCO_3_ was added to quench the reaction. The organic layer was washed with water for three times and dried with MgSO_4_. The crude product was concentrated via rotary evaporation and purified by column chromatography (petroleum ether: ethyl acetate = 10:1) to give the final product (yield: 46 %). ^1^H NMR (400 MHz, CDCl_3_, 298 K): δ 6.76 (s, 10H), 3.77 (s, 10H), 3.64 (s, 30H).


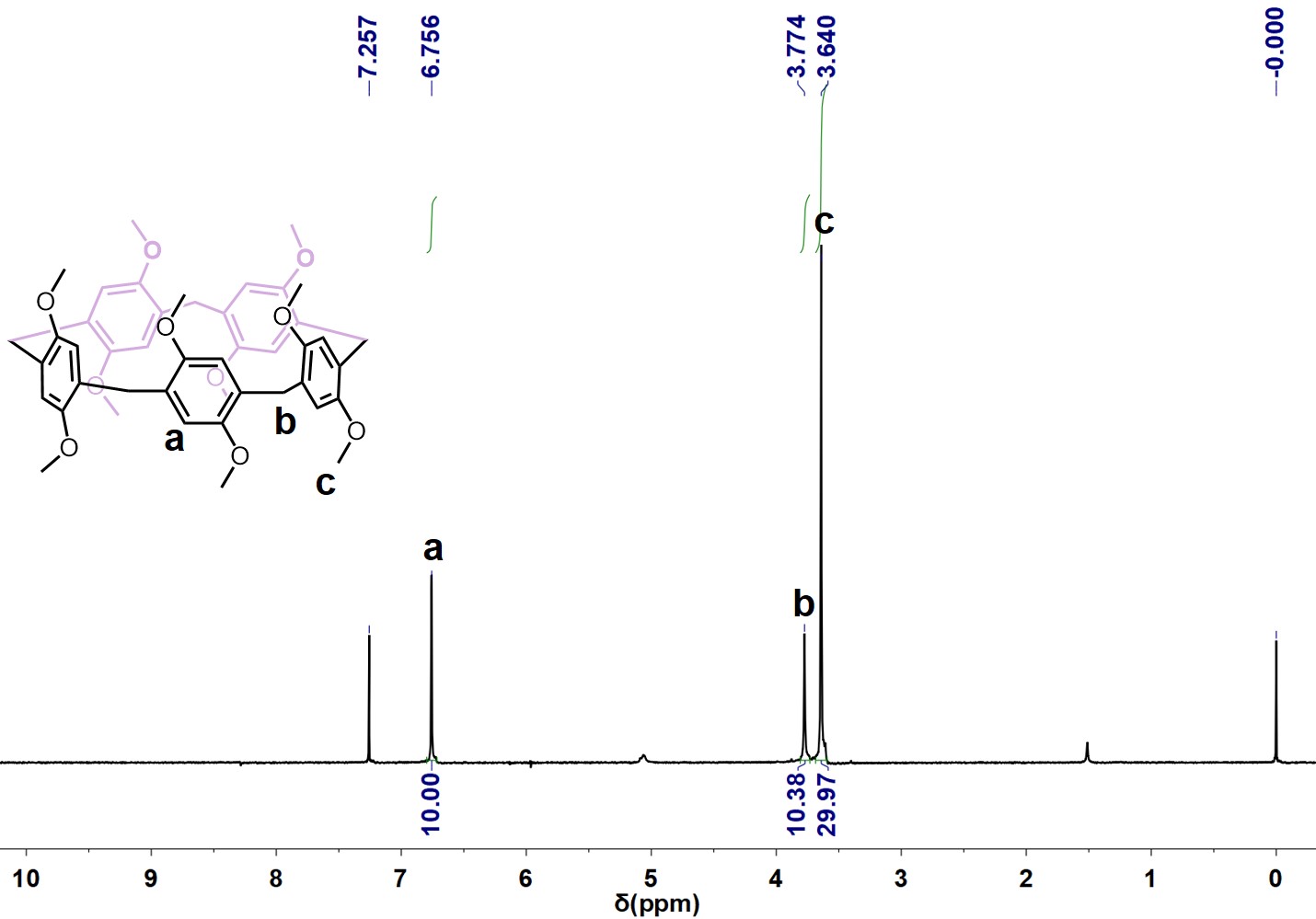


**Figure S2.** ^1^H NMR spectrum (400 MHz, CDCl_3_, 298 K) of compound **2**.

**Synthesis of compound 3:**

To a solution of compound **2** (2 g, 2.67 mmol) in dichloromethane (200 mL), an aqueous solution of ammonium ceric nitrate (2.92 g, 5.33 mmol) was added dropwise. After stirring the reaction mixture at room temperature for 1 h, the crude product was extracted with dichloromethane and then concentrated by rotary evaporation. A red powder of compound **3** was further obtained by silica gel column chromatography (petroleum ether: dichloromethane = 1:1) (yield, 77 %). ^1^H NMR (400 MHz, CDCl_3_, 298 K): δ 6.85 (s, 2H), 6.81 (s, 2H), 6.80 (s, 2H), 6.67 (s, 4H), 3.79 (s, 6H), 3.75 (s, 6H), 3.72 (s, 6H), 3.71 (s, 6H), 3.63 (s, 6H), 3.59 (s, 4H).

**
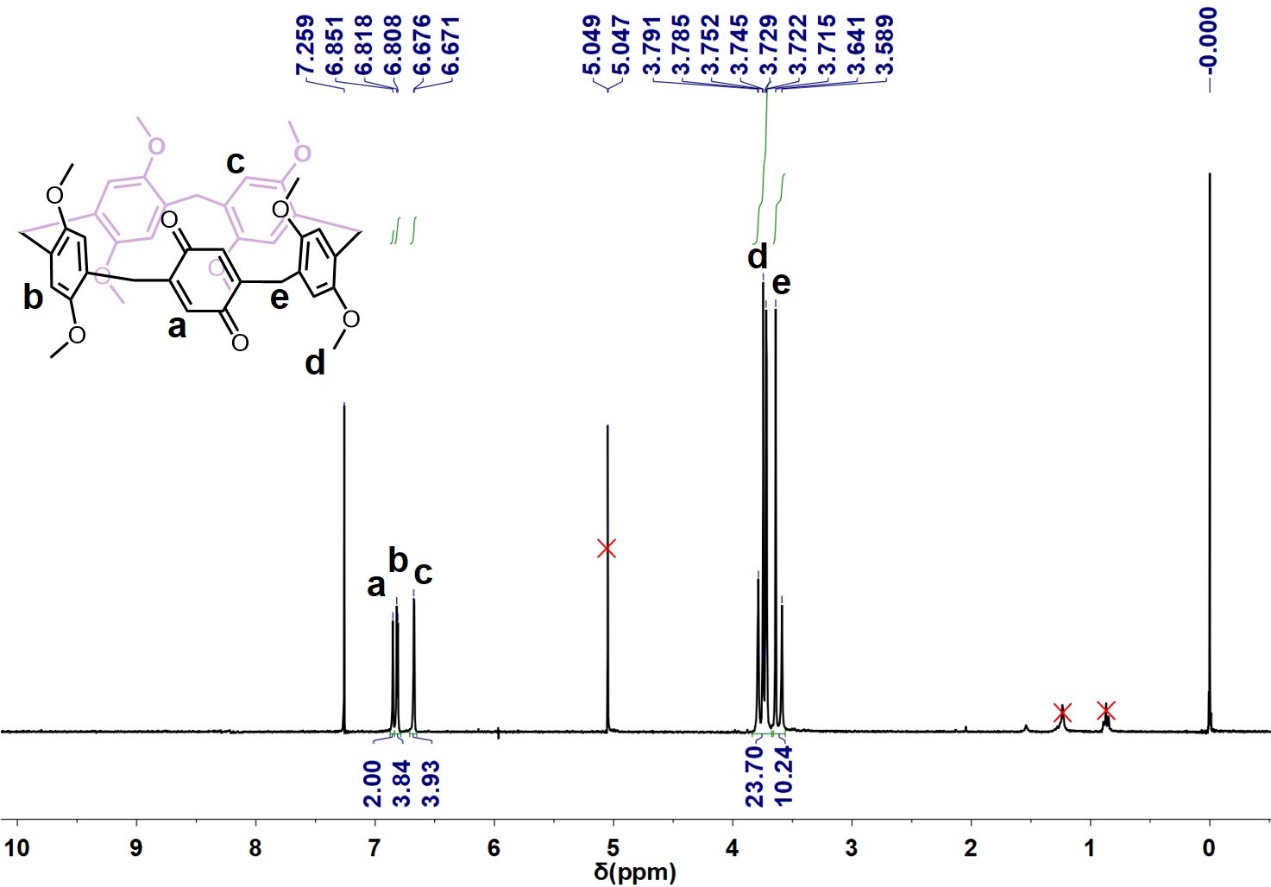
**

**Figure S3.** ^1^H NMR spectrum (400 MHz, CDCl_3_, 298 K) of compound **3**.

**Synthesis of Compound 5:**

Compound **3** was treated with an excess of Na_2_S_2_O_4_ in a mixed solvent of H_2_O and CH_2_Cl_2_ and the mixture was stirred until it turned pure white. The organic layer was concentrated to give a white product as compound **4**. Trifluoromethanesulfonic anhydride (4 mL) was then added dropwise to a mixture of **4** (1.6 g, 2 mmol) and pyridine (dry, 10 mL) under N_2_ atmosphere. The reaction mixture was allowed to stir at room temperature for 12 hours. The reaction was stopped and the mixture was extracted with DCM and washed with NaCl aqueous solution for 3 times. The organic extracts were concentrated by rotary evaporation and purified by column chromatography (petroleum: ethyl acetate = 10:1). Compound **5** was obtained as a white powder (yield, 65%). ^1^H NMR (400 MHz, CDCl_3_, 298 K): δ 7.33 (s, 2H), 6.79 (t, 6H), 6.67 (s, 2H), 3.85(s, 10H), 3.69 (m, 24H).


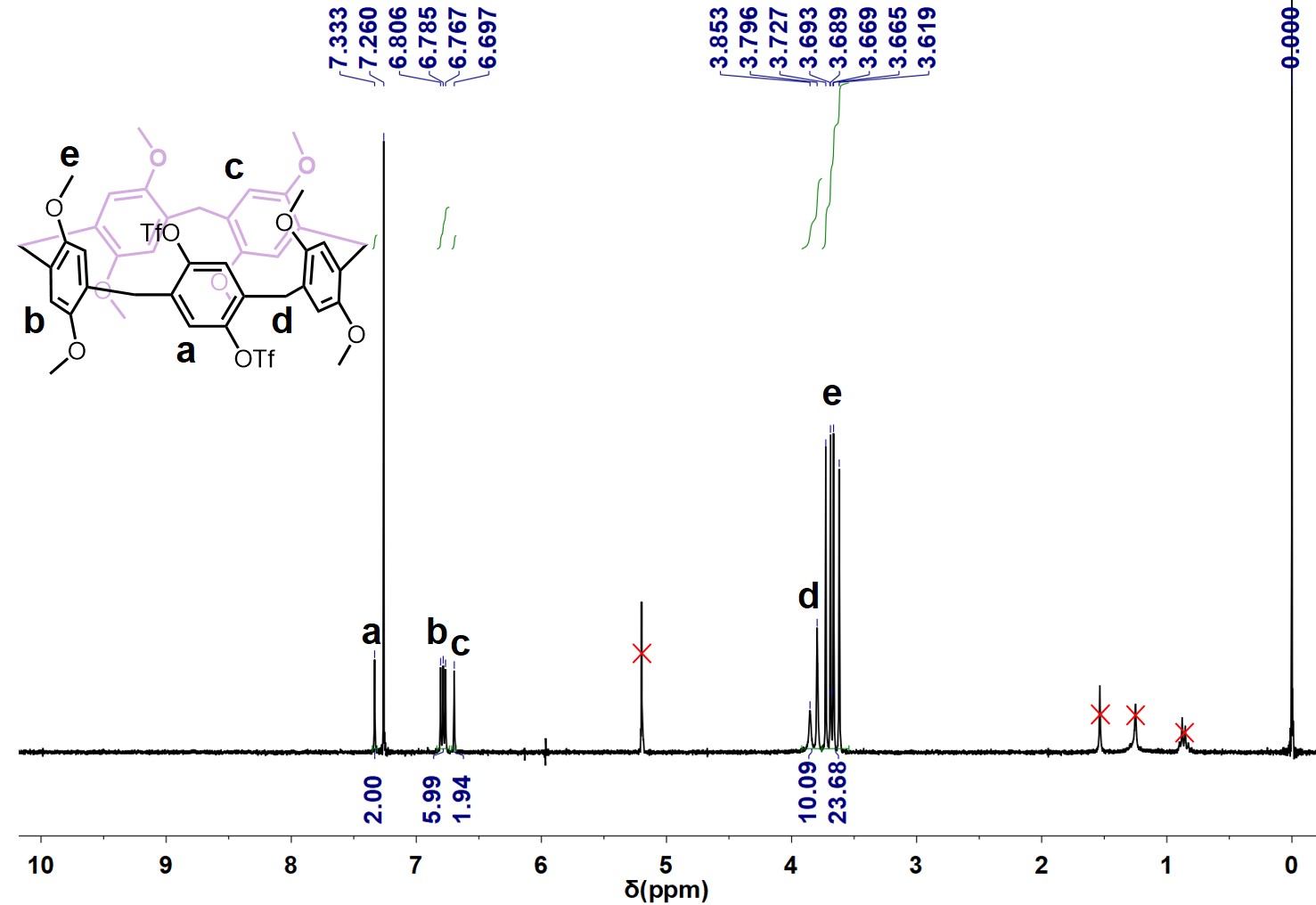


**Figure S4.** ^1^H NMR spectrum (400 MHz, CDCl_3_, 298 K) of compound **5**.

**Synthesis of L2:**

Compound **5** (1.4 g, 1.5 mmol), K_2_CO_3_ (2 g), Pd(PPh_3_)_2_Cl_2_ (200 mg), and pyridine-4-boronic acid (0.43 g, 3.5 mmol) were added into a 75 mL flask under nitrogen protection. Then THF (40 mL) and water (10 mL) were added into the mixture, which was heated at reflux for 72 hours. Then THF was removed by rotary evaporation, and CH_2_Cl_2_ was added to extract the organic components. The crude product was subjected to column chromatography (dichloromethane: MeOH=100:1) to produce the pure compound **L2** (yield, 50%). ^1^H NMR (400 MHz, CDCl_3_, 298 K): 8.43 (d, 4H), 6.88 (t, 6H), 6.83 (s, 2H), 6.73 (s, 2H), 6.54 (s, 2H), 5.95 (s, 2H), 3.73 (m, 34H).


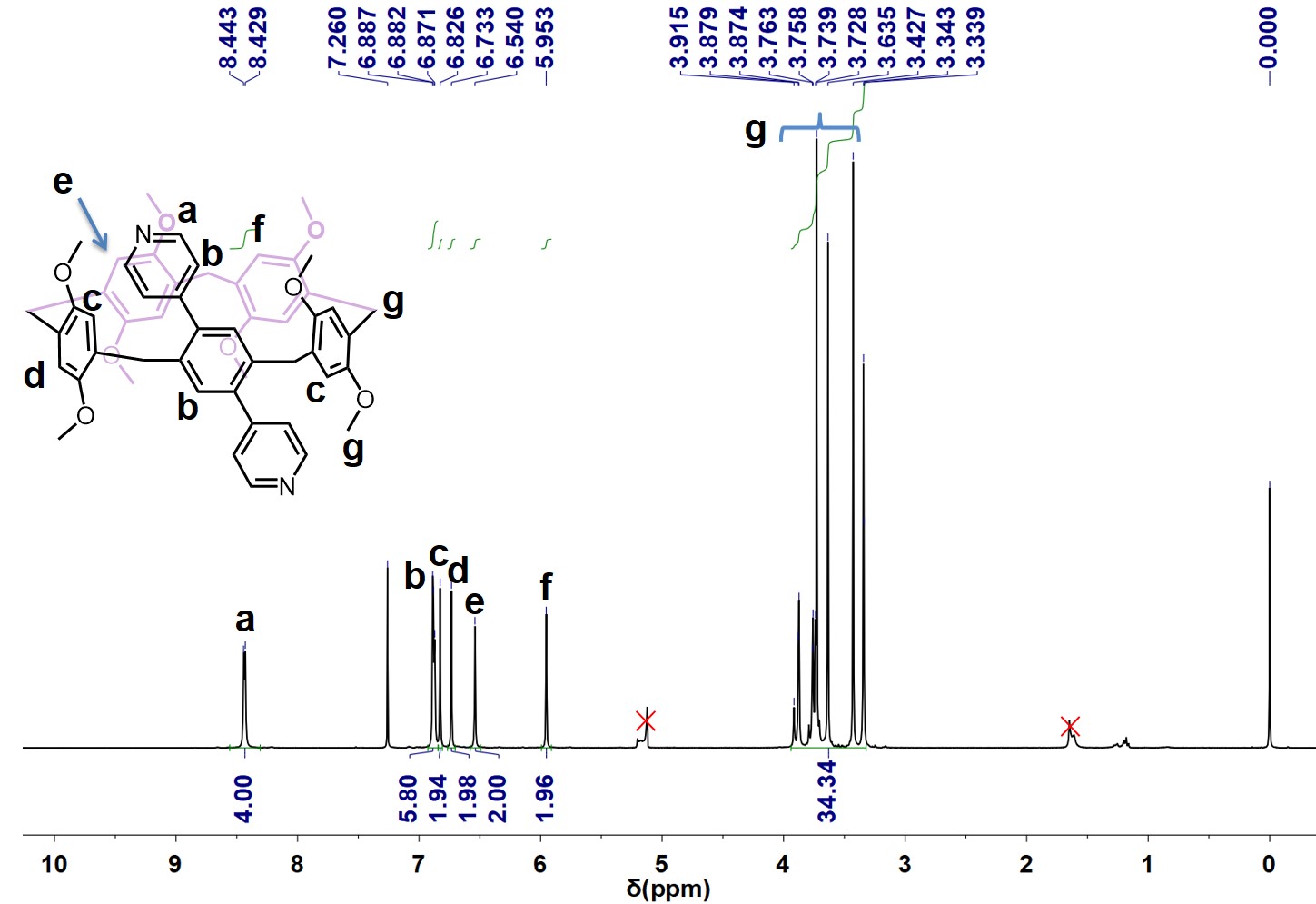


**Figure S5.** ^1^H NMR spectrum (400 MHz, CDCl_3_, 298 K) of **L2**.

**
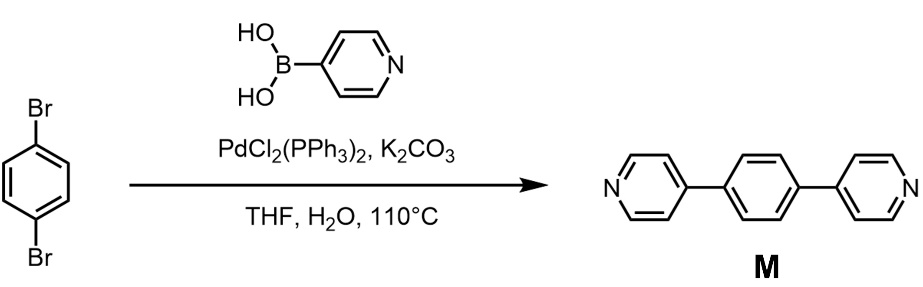
**

**Scheme S3.** Synthetic route of **M**.

**Synthesis of M:**

4-Pyridinylboronic acid (1.25 g, 10 mmol), 1,4-dibromobenzene (1 g, 4.2 mmol), and K_2_CO_3_ (2.8 g, 20.4 mmol) were added to a mixture of THF (40 mL) and H_2_O (20 mL). Next, Pd(PPh_3_)_4_ (0.39 g, 0.34 mmol) was added and the mixture was heated at 130 °C under N_2_ protection for 72 h. Then the reaction mixture was cooled to room temperature and filtered. The organic phase was concentrated via rotary evaporation and the residue was dissolved in CHCl_3_ and washed three times with water. The collected organic phase was concentrated under vacuum and the residue was dissolved in acetone. Concentrated HCl was then added dropwise to the solution, and the resulting precipitate was collected by filtration and then dissolved in H_2_O. Finally, aqueous NaOH (10 M) was added dropwise until the pH was ca. 8-9 to obtain the product **M** as a white powder (yield, 80 %). ^1^H NMR (400 MHz, CDCl_3_, 298 K): δ 8.70 (q, 4H), 7.78 (s, 4H), 7.56 (q, 4H).


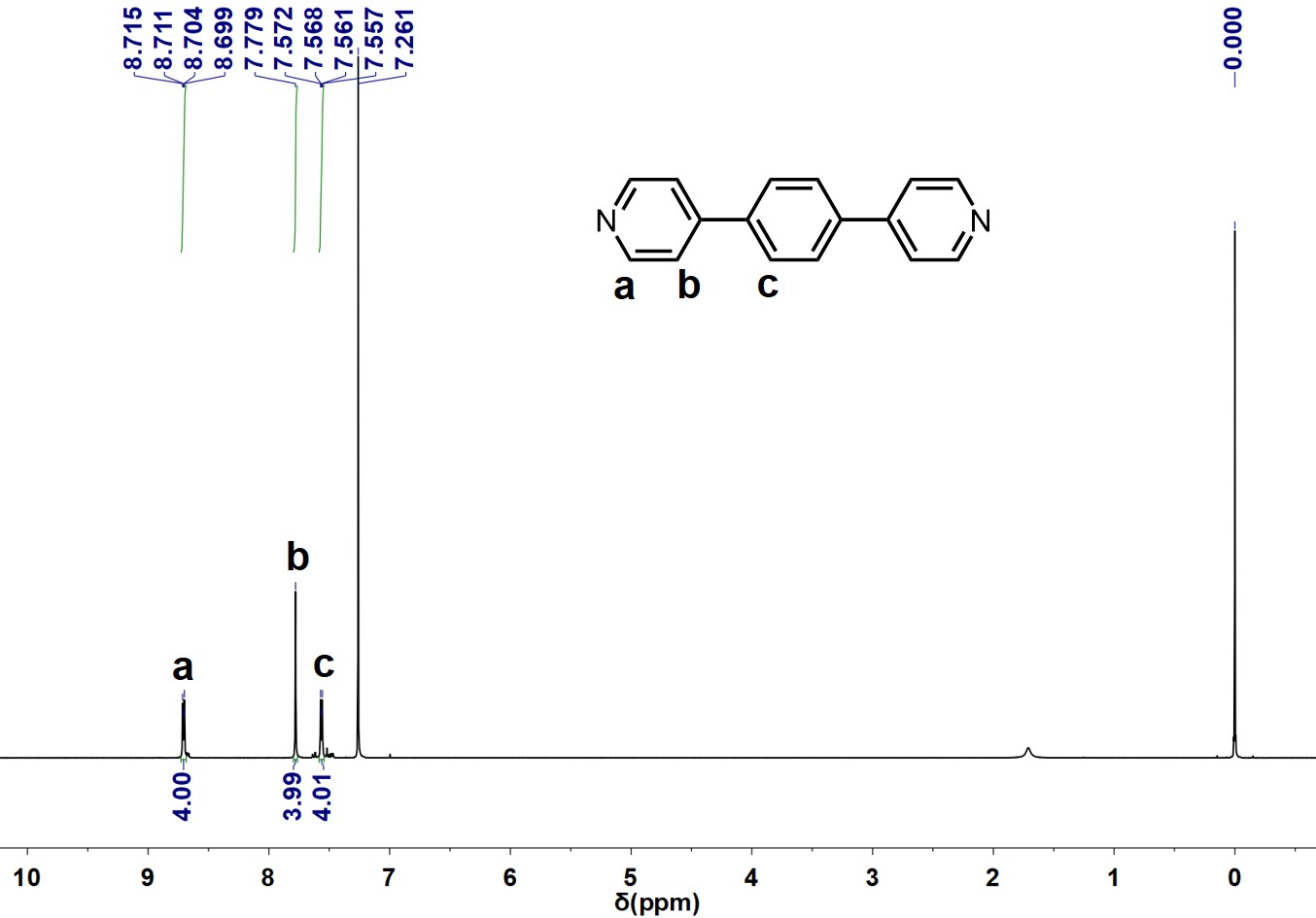


**Figure S6.** ^1^H NMR spectrum (400 MHz, CDCl_3_, 298 K) of **M**.

## 2. Preparation of the materials

**Synthesis of PHM:**

CdCl_2_•2.5H_2_O (22.8 mg, 0.1mmol), L1 (24.8 mg, 0.1 mmol), and L2 (84.5 mg, 0.1 mmol) were dissolved in a mixture of DMF (8 mL) and isopropanol (2 mL) in a 25 mL Teflon tube. Then the solution was heated under high pressure at 120 °C for 48 hours. The crude product was separated by centrifugation and washed with DMF, ethanol, and water, respectively, for several times to give a yellow non-soluble powder as PHM.

**Synthesis of MHM:**

CdCl_2_•2.5H_2_O (22.8 mg, 0.1mmol), L1 (24.8 mg, 0.1 mmol) and M (23.2 mg, 0.1 mmol) were dissolved in a mixture of 8 mL DMF and 2 mL isopropanol in a 25 mL Teflon. Then the solution was heated at a temperature of 120°C under high pressure for 48 hours. The crude product was separated by centrifugation and washed with DMF, ethanol, and water respectively for several times to give a yellow non-soluble powder as MHM.

## 3. Structural analysis


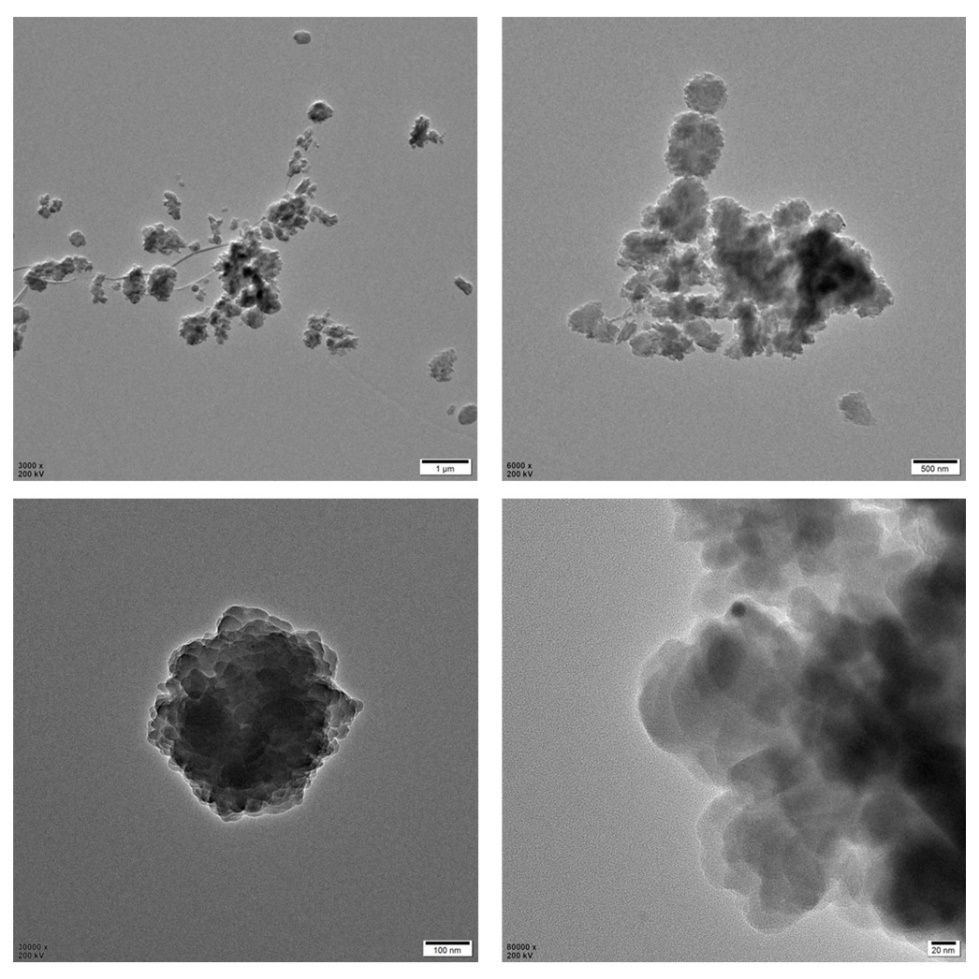


**Figure S7.** TEM images of PHM.


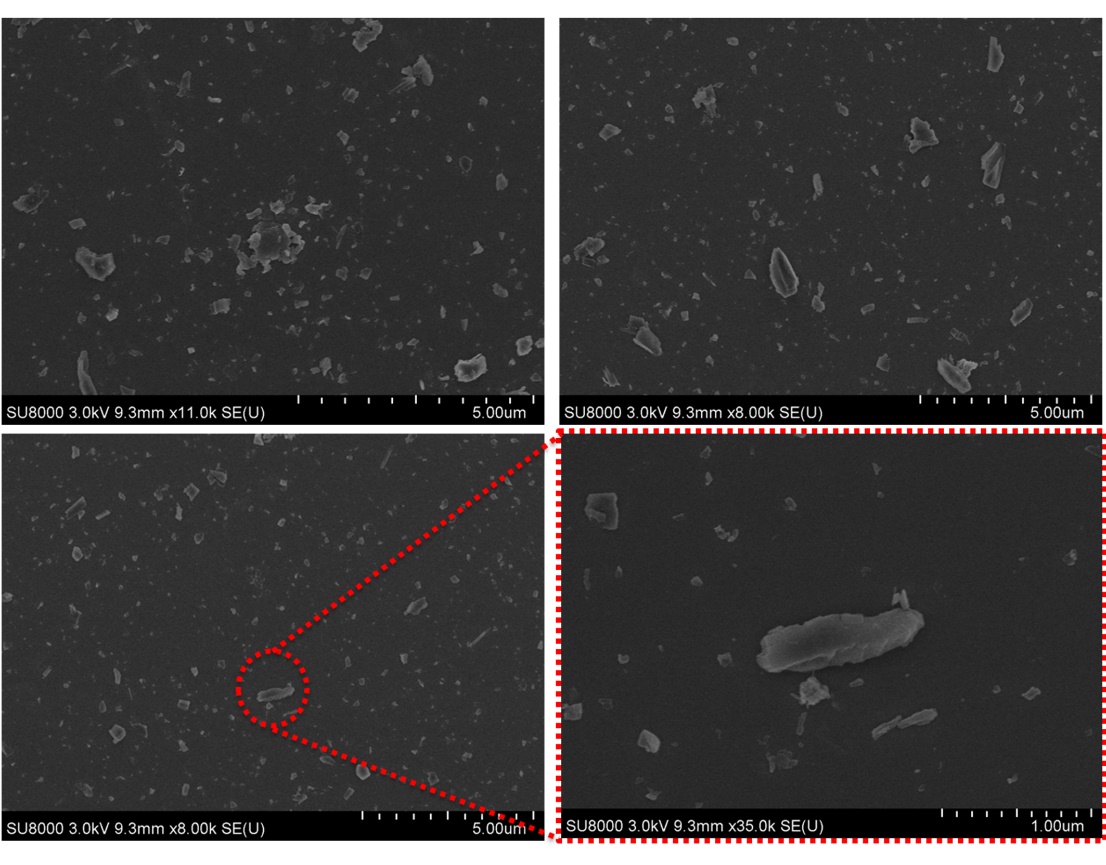


**Figure S8.** SEM images of MHM.


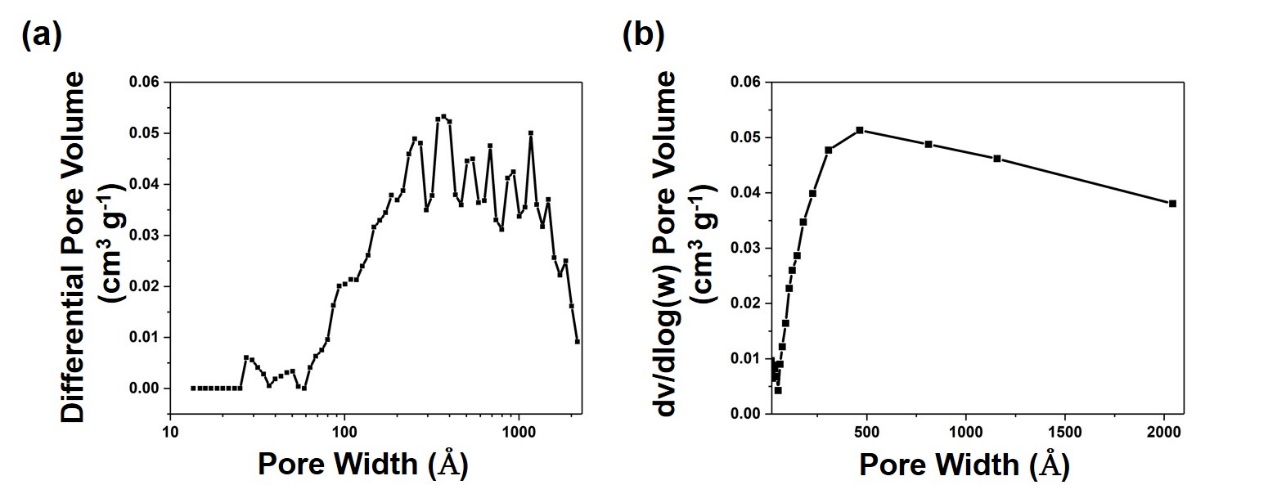


**Figure S9.** (a) Differential pore volume and (b) the pore size distribution analysis of PHM.


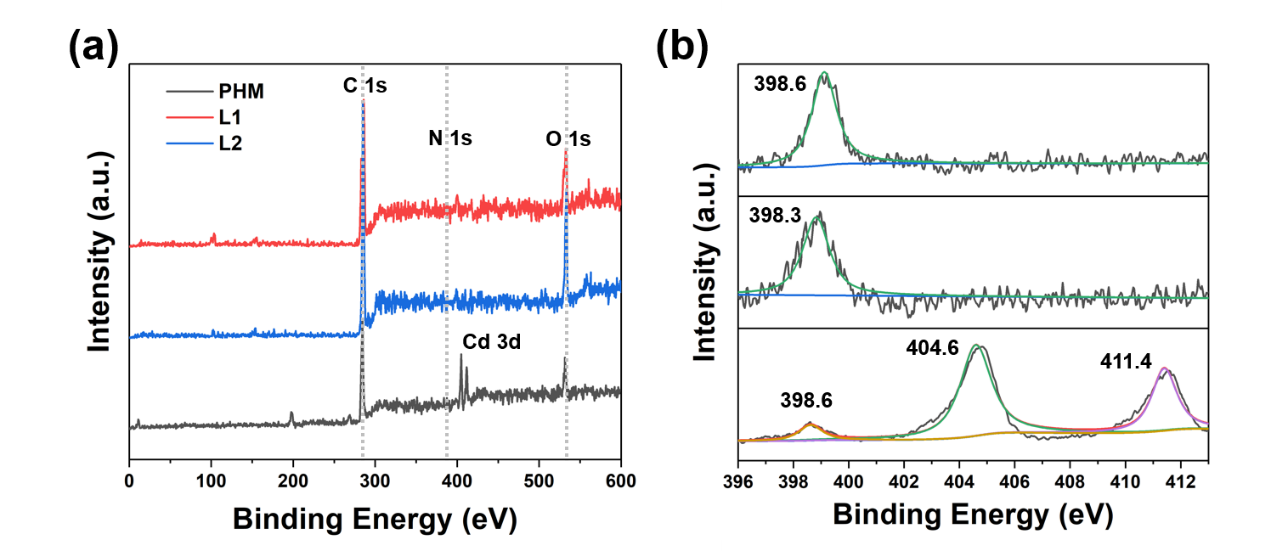


**Figure S10.** (a) XPS wide-range spectra of PHM (black), L1 (red), and L2 (blue). (b) Partial spectra (396-413 eV) including N 1s and Cd 3d regions of L1, L2 and PHM.


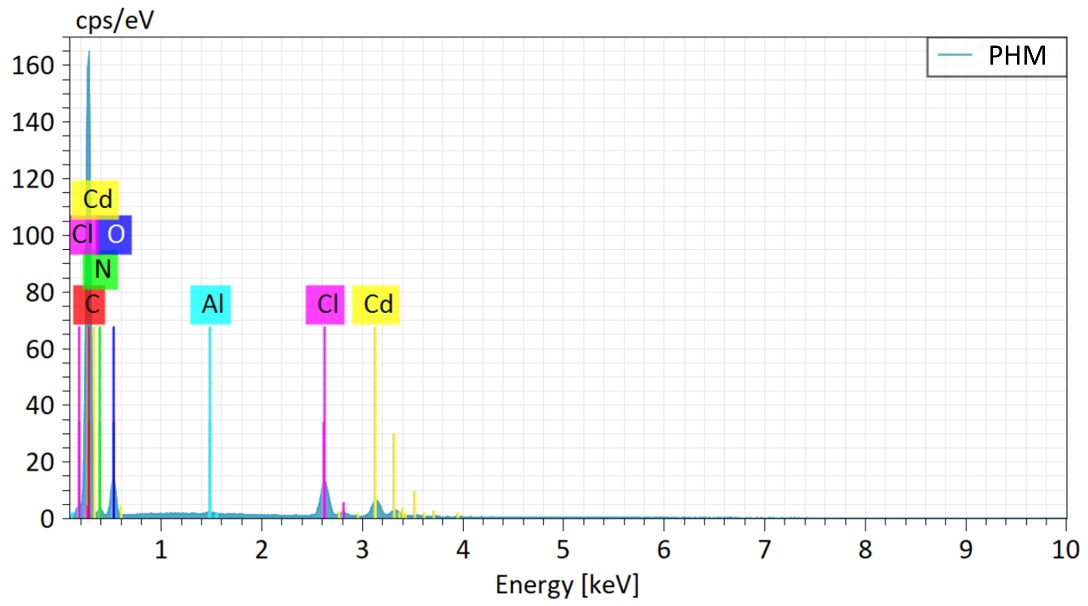


**Figure S11.** Energy spectrum of PHM.

**
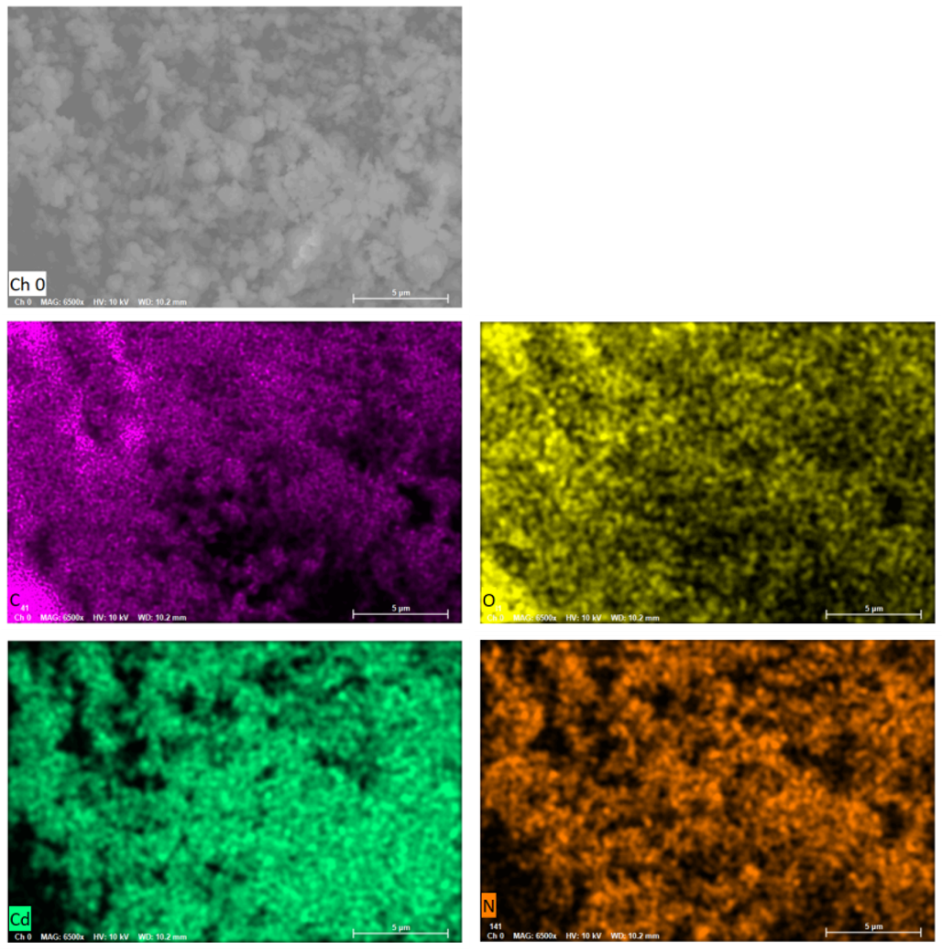
**

**Figure S12.** EDS mapping images of PHM containing the elements of C, O, Cd, and N.

**Table S1.** Atomic ratios of each element in PHM

| Element | C | Cd | Cl | O | N |
| --- | --- | --- | --- | --- | --- |
| Atomic ratio (%) | 80.26 | 2.17 | 4.11 | 7.54 | 5.82 |

**Table S2.** Ligand ratio of L1/L2

|  | Molecular weight (g mol^-1^) | Weight ratio | Ligand ratio |
| --- | --- | --- | --- |
| L1 | 284 | 0.3 | 1.05 |
| L2 | 845 | 0.5 | 0.59 |

## 4. Fluorescence data of PHM


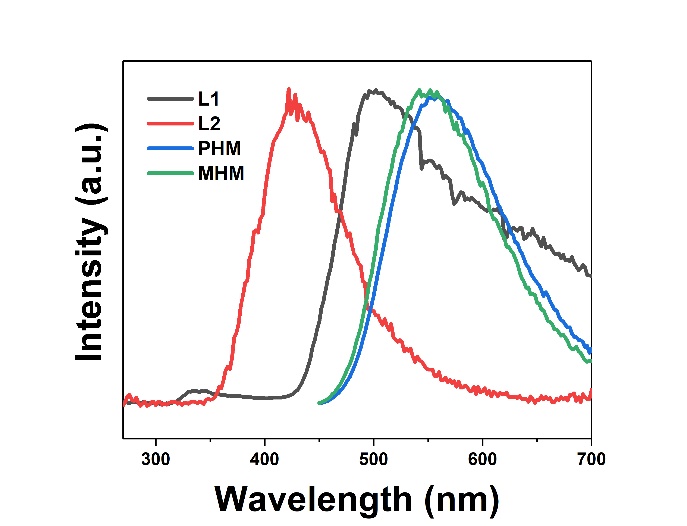


**Figure S13.** Solid-state fluorescence spectra of L1, L2, PHM, and MHM.


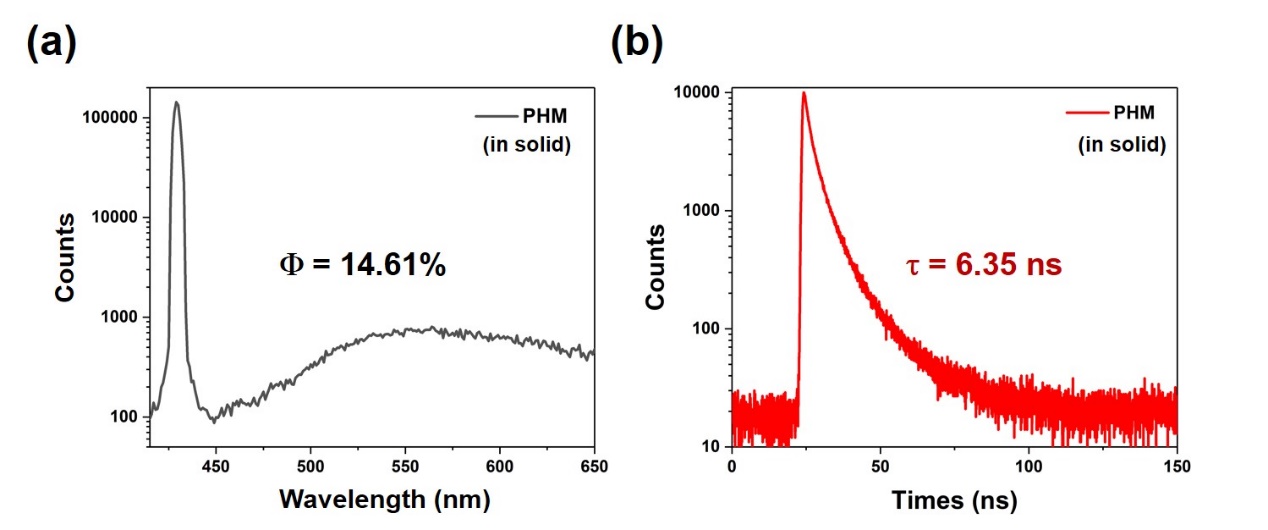


**Figure S14.** (a) Quantum yield and (b) fluorescence lifetime of PHM in the solid state.


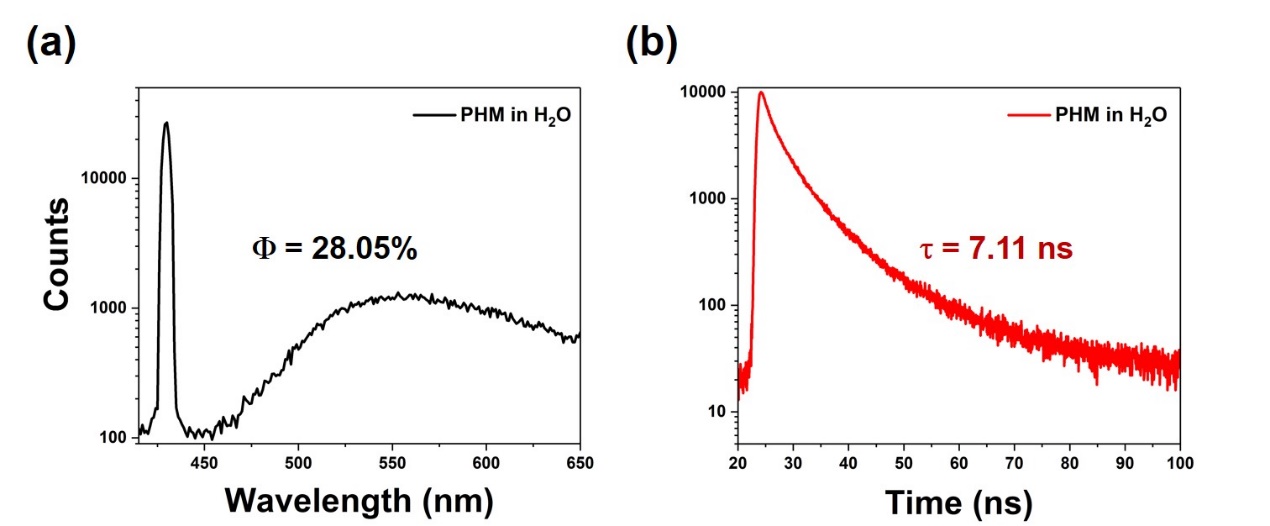


**Figure S15.** (a) Quantum yield and (b) fluorescence lifetime of PHM in H_2_O.

## 5. DFT calculation


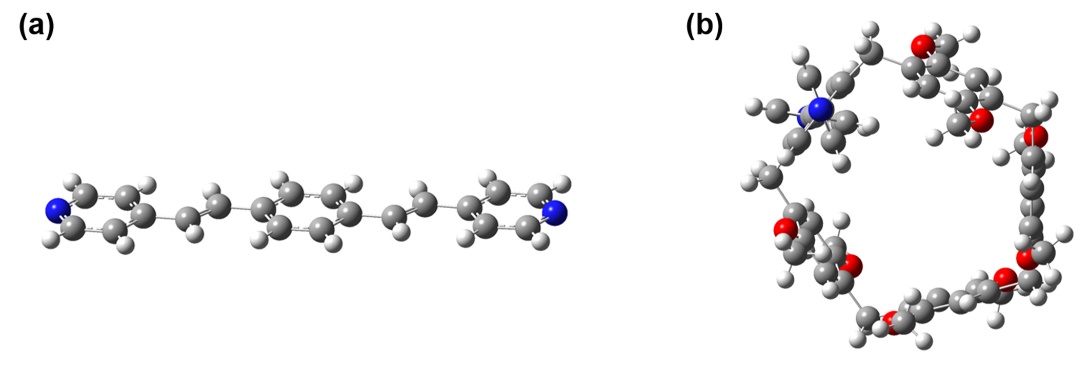


**Figure S16.** Optimized ball-and-stick model of (a) L1 and (b) L2.

**Table S3.** Coordinates (Å) for the optimized structure (B3LYP, 3-21G*) of L1

| C | -1.13122 | -0.74661 | 0 |
| --- | --- | --- | --- |
| C | 0.263938 | -0.74661 | 0 |
| C | 0.96148 | 0.46114 | 0 |
| C | 0.26382 | 1.66965 | -0.0012 |
| C | -1.131 | 1.66958 | -0.00168 |
| H | -1.68098 | -1.69892 | 4.50E-04 |
| H | 0.81345 | -1.69912 | 0.00132 |
| H | 0.81402 | 2.6218 | -0.00126 |
| H | -1.68112 | 2.62186 | -0.00263 |
| C | 2.50148 | 0.46126 | 8.88E-04 |
| H | 3.03471 | -0.46641 | 0.0015 |
| C | 3.18363 | 1.63225 | 8.91E-04 |
| H | 2.65592 | 2.56307 | -6.00E-05 |
| C | 4.72361 | 1.62321 | 0.00227 |
| C | 5.42821 | 2.82738 | 0.00228 |
| C | 5.41401 | 0.41124 | 0.00362 |
| C | 6.8229 | 2.81947 | 0.00295 |
| H | 4.88361 | 3.78271 | 4.31E-04 |
| C | 6.80912 | 0.40315 | 0.00529 |
| H | 4.85883 | -0.53793 | 0.0038 |
| C | 7.51362 | 1.60699 | 0.00482 |
| H | 7.37834 | 3.76856 | 0.00232 |
| H | 7.35321 | -0.55259 | 0.00678 |
| C | 9.0536 | 1.59861 | 0.00565 |
| H | 9.1829 | 0.53645 | 0.00704 |
| C | 10.13837 | 2.41092 | 0.00522 |
| H | 10.01539 | 3.47382 | 0.00384 |
| C | 11.55292 | 1.80211 | 0.00673 |
| C | 12.67129 | 2.63621 | 0.00629 |
| C | 11.71609 | 0.41686 | 0.00864 |
| C | 13.9525 | 2.0851 | 0.00708 |
| H | 12.54231 | 3.72828 | 0.004 |
| C | 12.99762 | -0.13458 | 0.01043 |
| H | 10.83475 | -0.24069 | 0.00917 |
| H | 14.83405 | 2.74248 | 0.00611 |
| H | 13.12596 | -1.22683 | 0.01236 |
| N | 14.11576 | 0.69926 | 0.00952 |
| N | -1.8286 | 0.46137 | -6.82E-04 |

**Table S4.** Coordinates (Å) for the optimized structure (B3LYP, 3-21G*) of L2

| C | -2.43336 | -2.9646 | 1.402932 |
| --- | --- | --- | --- |
| C | -1.78157 | -3.73598 | 0.449711 |
| H | -2.21992 | -3.93969 | -0.34394 |
| C | -0.48395 | -4.21522 | 0.65361 |
| C | 0.19226 | -3.93553 | 1.84048 |
| C | -0.47281 | -3.16842 | 2.79876 |
| H | -0.04266 | -2.97549 | 3.6003 |
| C | -1.75581 | -2.68282 | 2.59287 |
| C | 1.60645 | -4.42277 | 2.07185 |
| H | 1.75176 | -4.5494 | 3.02198 |
| H | 1.72654 | -5.27949 | 1.6347 |
| C | 2.62399 | -3.44062 | 1.53686 |
| C | 3.16925 | -3.59431 | 0.26559 |
| H | 2.92794 | -4.33269 | -0.24668 |
| C | 4.07268 | -2.6624 | -0.25615 |
| C | 4.44647 | -1.55313 | 0.50089 |
| C | 3.89371 | -1.40433 | 1.76393 |
| H | 4.12382 | -0.65959 | 2.27317 |
| C | 3.00995 | -2.33187 | 2.28809 |
| C | 5.42363 | -0.51574 | -0.04041 |
| H | 6.04275 | -0.25646 | 0.66038 |
| H | 5.93787 | -0.90326 | -0.768 |
| C | 4.68334 | 0.71457 | -0.54322 |
| C | 4.11528 | 0.6997 | -1.8128 |
| H | 4.22227 | -0.05141 | -2.35107 |
| C | 3.39205 | 1.78035 | -2.29605 |
| C | 3.21123 | 2.91281 | -1.50546 |
| C | 3.78959 | 2.92613 | -0.23665 |
| H | 3.67765 | 3.67452 | 0.30527 |
| C | 4.52669 | 1.8511 | 0.23659 |
| C | 2.36458 | 4.08131 | -1.98434 |
| H | 2.63415 | 4.88446 | -1.51258 |
| H | 2.52413 | 4.22338 | -2.93123 |
| C | 0.88485 | 3.84287 | -1.75819 |
| C | 0.14845 | 3.10738 | -2.6839 |
| H | 0.57048 | 2.79074 | -3.44899 |
| C | -1.20039 | 2.83636 | -2.49135 |
| C | -1.85705 | 3.30878 | -1.35585 |
| C | -1.12451 | 4.06346 | -0.43814 |
| H | -1.54751 | 4.39914 | 0.31894 |
| C | 0.23149 | 4.3237 | -0.63531 |
| C | -3.3274 | 2.98728 | -1.11269 |
| H | -3.70345 | 3.65622 | -0.52012 |
| H | -3.80417 | 3.03813 | -1.95514 |
| C | -3.54187 | 1.61516 | -0.50569 |
| C | -3.6705 | 0.49988 | -1.33406 |
| H | -3.65518 | 0.63397 | -2.25407 |
| C | -3.81994 | -0.80441 | -0.86027 |
| C | -3.81107 | -1.02583 | 0.53744 |
| C | -3.6977 | 0.08185 | 1.36122 |
| H | -3.70562 | -0.05253 | 2.28119 |
| C | -3.5721 | 1.38276 | 0.8884 |
| C | -3.04868 | -2.07908 | -2.85958 |
| H | -2.28065 | -1.55771 | -2.88734 |
| C | -3.25199 | -3.05964 | -3.81087 |
| H | -2.59428 | -3.18105 | -4.45726 |
| C | -5.23384 | -3.63816 | -2.92135 |
| H | -5.99707 | -4.17059 | -2.92624 |
| C | -5.12102 | -2.68163 | -1.91932 |
| H | -5.79723 | -2.57987 | -1.28954 |
| C | -3.98479 | -1.87652 | -1.86822 |
| C | -4.39094 | 3.50794 | 1.97822 |
| H | -5.12574 | 3.53151 | 1.40815 |
| C | -4.22263 | 4.50527 | 2.93764 |
| H | -4.86658 | 5.17551 | 2.98972 |
| C | -2.3115 | 3.57441 | 3.67166 |
| H | -1.58129 | 3.59772 | 4.24518 |
| C | -2.39369 | 2.51826 | 2.76573 |
| H | -1.74663 | 1.84963 | 2.75705 |
| C | -3.46537 | 2.48576 | 1.87461 |
| C | -3.82541 | -2.423 | 1.14646 |
| H | -4.31526 | -2.39829 | 1.98316 |
| H | -4.29365 | -3.02449 | 0.54665 |
| C | -0.40308 | -5.31321 | -1.47471 |
| H | -1.1636 | -5.87295 | -1.29833 |
| H | 0.22214 | -5.78748 | -2.02842 |
| H | -0.6915 | -4.5161 | -1.92484 |
| C | -1.72939 | -1.50095 | 4.68263 |
| H | -1.3846 | -2.27257 | 5.13885 |
| H | -2.32907 | -1.02635 | 5.26383 |
| H | -1.00271 | -0.92588 | 4.43327 |
| C | 4.71671 | -4.00669 | -2.08355 |
| H | 4.83871 | -4.65873 | -1.39084 |
| H | 5.47417 | -4.01886 | -2.67287 |
| H | 3.91937 | -4.21233 | -2.5797 |
| C | 2.5185 | -0.98965 | 4.21419 |
| H | 3.42613 | -0.79713 | 4.46434 |
| H | 1.97071 | -1.02021 | 5.00122 |
| H | 2.19713 | -0.30398 | 3.62471 |
| C | 2.92208 | 0.67784 | -4.36567 |
| H | 3.84234 | 0.493 | -4.57244 |
| H | 2.43468 | 0.82466 | -5.17934 |
| H | 2.5423 | -0.06967 | -3.89601 |
| C | 5.40872 | 3.0462 | 2.11762 |
| H | 5.89696 | 3.59699 | 1.50097 |
| H | 5.94632 | 2.88219 | 2.89573 |
| H | 4.6008 | 3.49507 | 2.37676 |
| C | -1.35079 | 1.51127 | -4.50654 |
| H | -0.9954 | 2.19223 | -5.08248 |
| H | -1.99501 | 0.98733 | -4.98902 |
| H | -0.63755 | 0.94343 | -4.20468 |
| C | 0.29692 | 5.85147 | 1.19099 |
| H | -0.35957 | 6.37916 | 0.73219 |
| H | 0.91668 | 6.43184 | 1.6395 |
| H | -0.13833 | 5.28806 | 1.83525 |
| N | -4.33419 | -3.8466 | -3.8672 |
| N | -3.21498 | 4.55854 | 3.77305 |
| O | 0.20615 | -4.97262 | -0.27487 |
| O | -2.42828 | -1.91331 | 3.5169 |
| O | 4.59484 | -2.76891 | -1.52642 |
| O | 2.46709 | -2.23745 | 3.55601 |
| O | 2.84438 | 1.82663 | -3.55926 |
| O | 5.0898 | 1.82147 | 1.50252 |
| O | -1.98167 | 2.12111 | -3.37874 |
| O | 0.98972 | 5.05279 | 0.26207 |

## 6. Luminescent responses of PHM


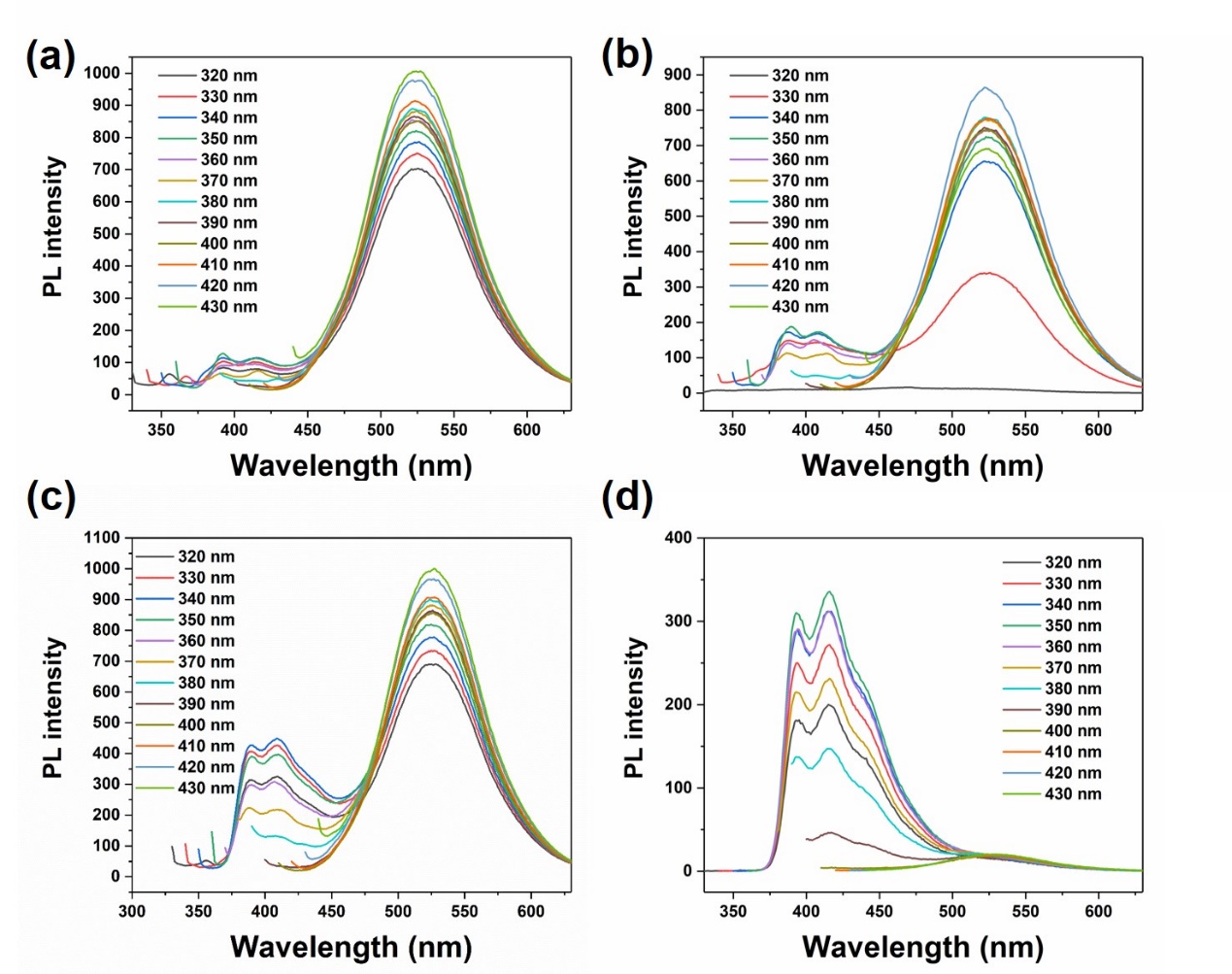


**Figure S17.** PL spectra of PHM suspension in (a) ethanol, (b) acetone, (c) acetonitrile, and (d) DMF, with the excitation wavelengths varying from 320 nm to 430 nm.


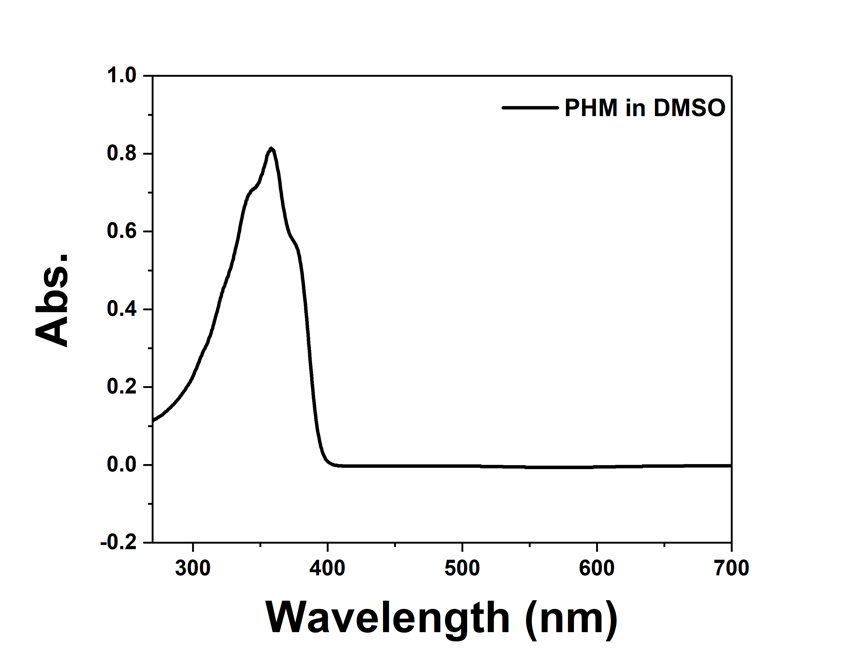


**Figure S18.** UV-vis absorption spectrum of PHM in DMSO.


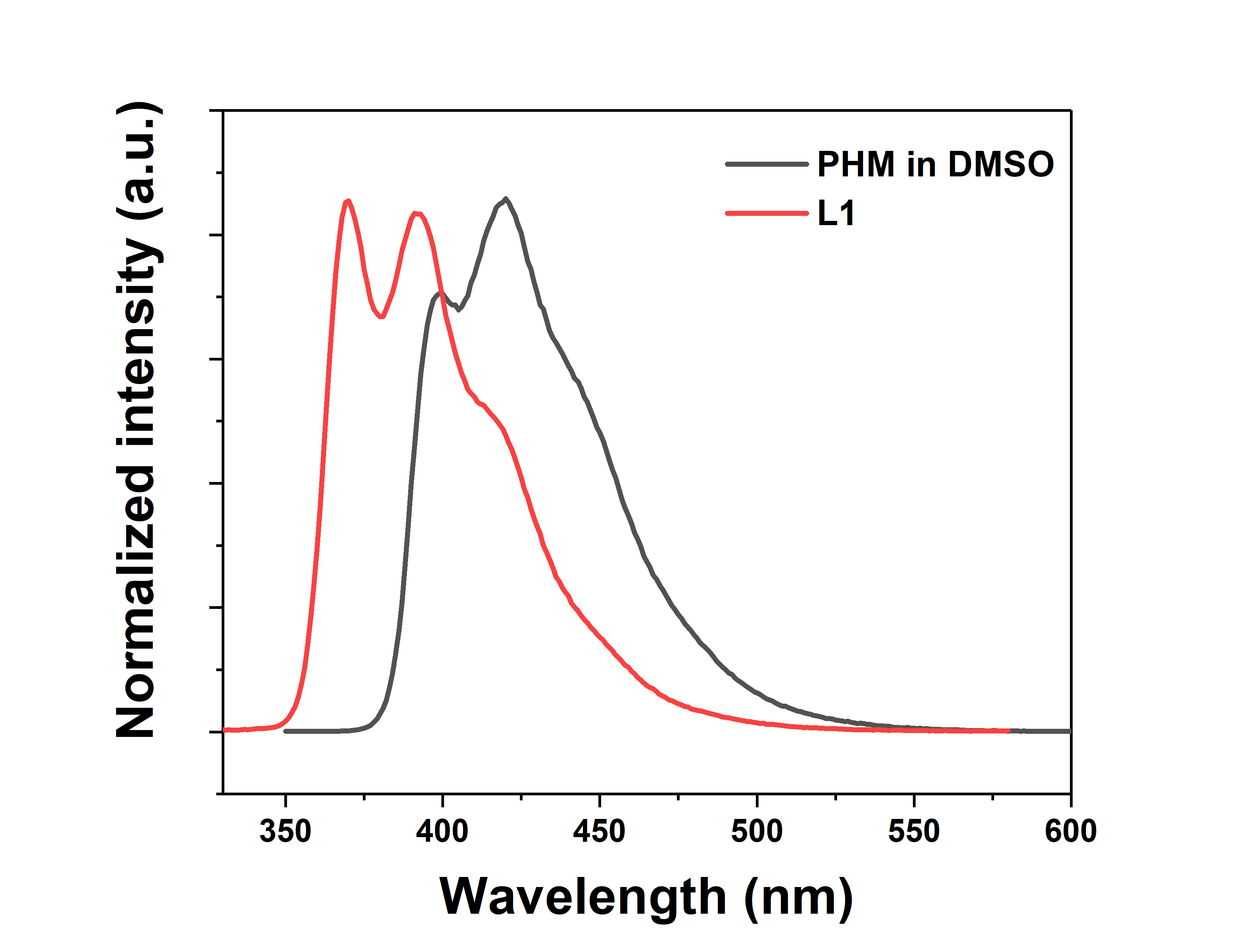


**Figure S19.** Normalized fluorescence spectra of PHM in DMSO (black) and L1 (red).


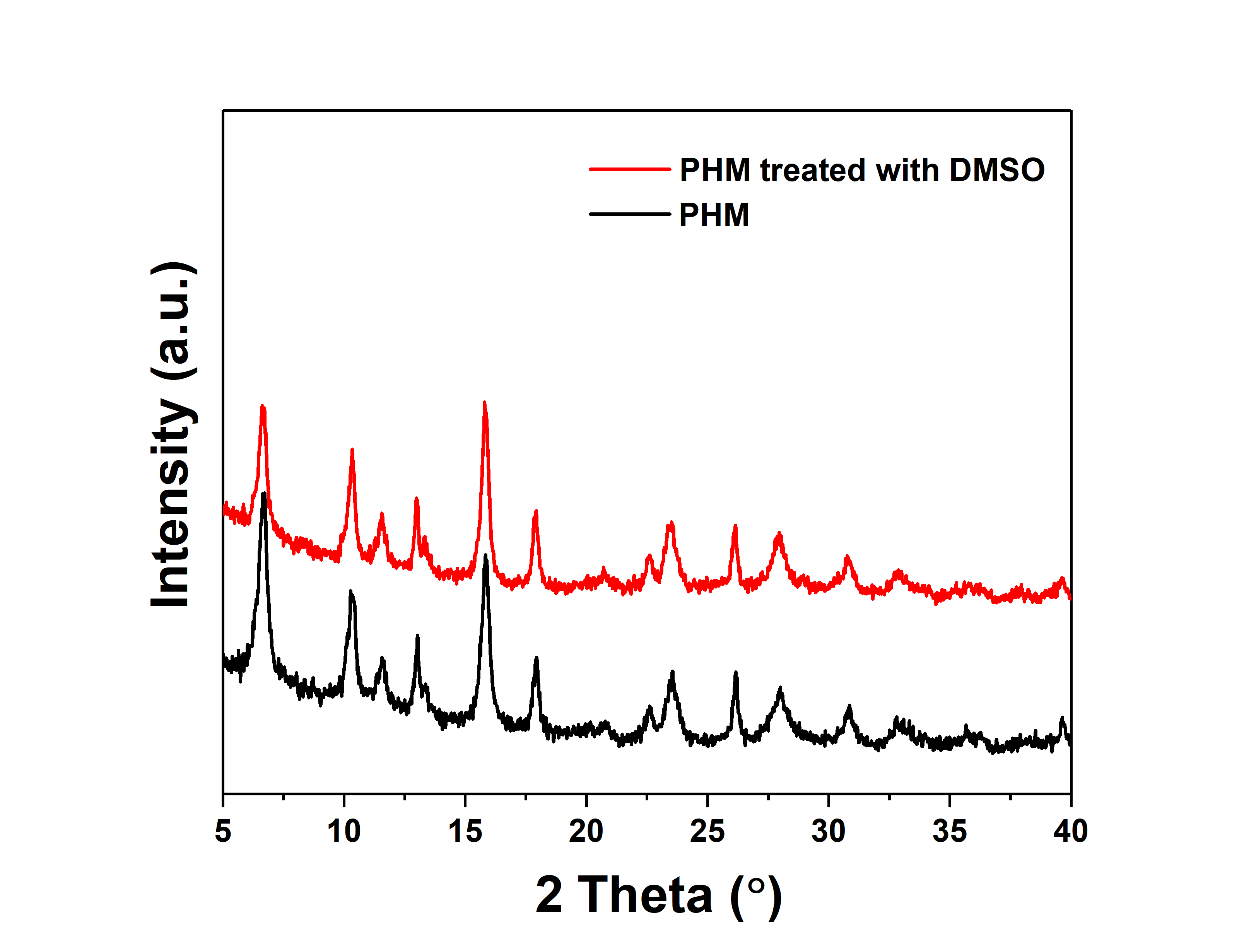


**Figure S20.** PXRD patterns of PHM before and after treatment with DMSO.


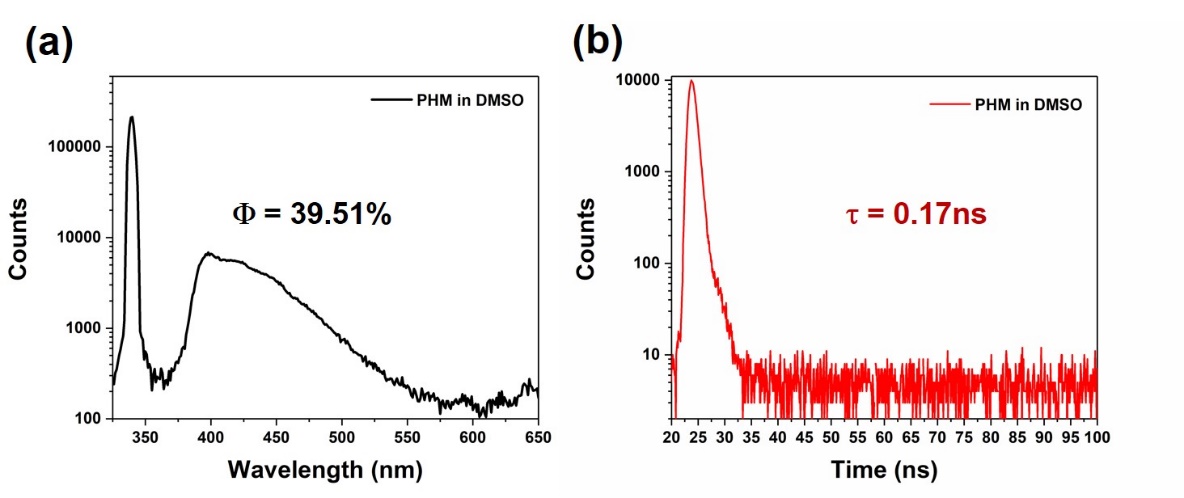


**Figure S21.** The quantum yield (a) and fluorescence lifetime (b) of PHM in DMSO.


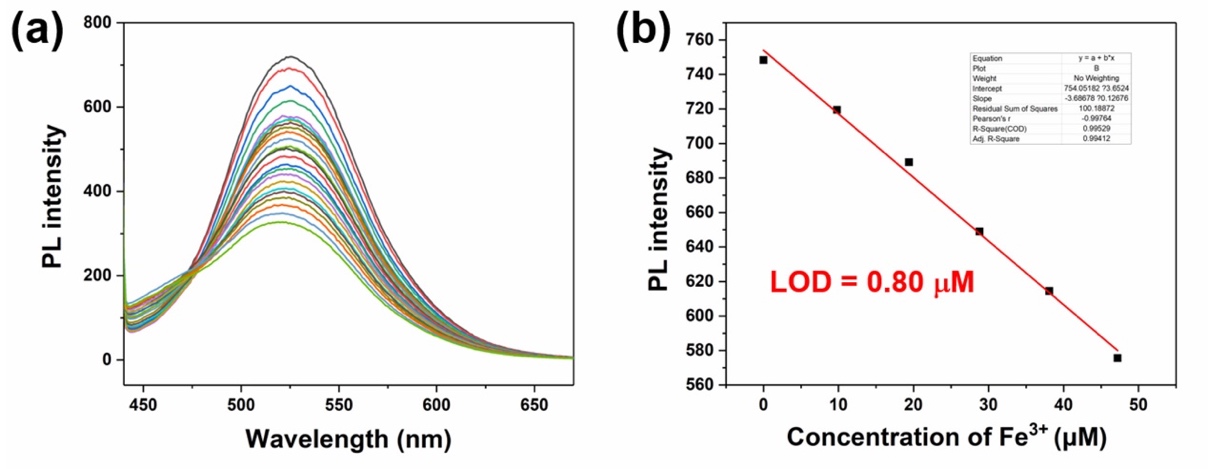


**Figure S22.** (a) PL spectra of PHM suspension in water ([PHM] = 10 μg mL^-1^) upon gradual addition of Fe^3+^ aqueous solutions ([Fe^3+^] = 10^-3^ M, V = 0~480 μL). λ_ex_ = 430 nm; slit width: Ex. 5 nm, Em. 5 nm. (b) Linear relationship between PL intensities at 530 nm and the concentration of Fe^3+^, and the limit of detection of PHM towards Fe^3+^ in water, whereby the LOD was calculated via 3SD/S rule (SD = standard deviation of 3-blank reading of PHM fluorescence intensity, S = slope of concentration of Fe^3+^ versus fluorescence intensity, concentration range 0 ‒ 50 µM).


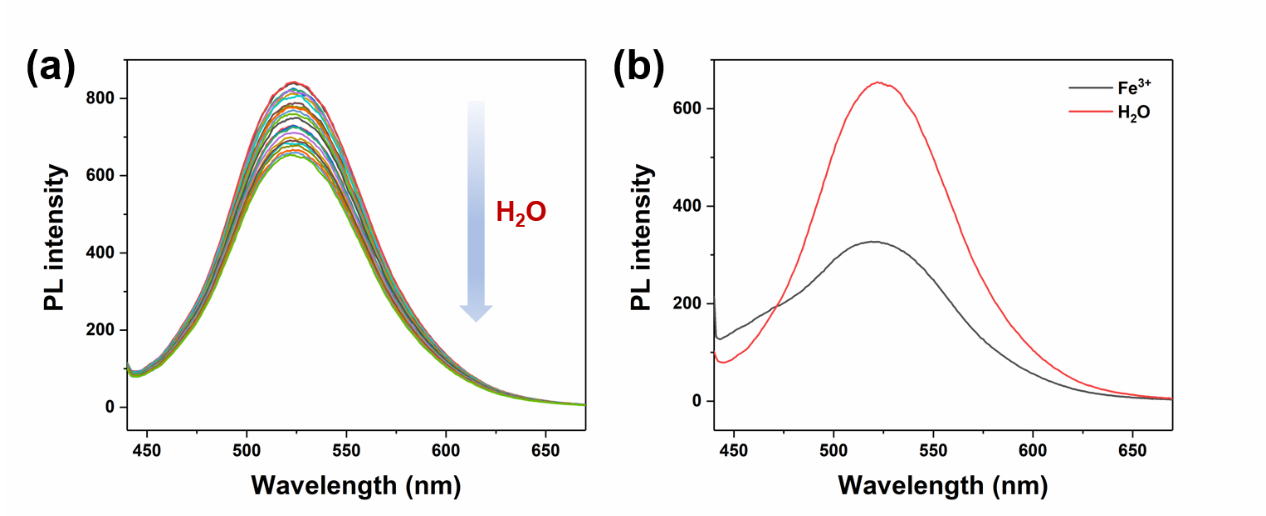


**Figure S23.** (a) Fluorescence titration of PHM water suspension ([PHM] = 10 μg mL^-1^) with deionized water. (b) PL spectra of PHM water suspension (PHM] = 10 μg mL^-1^) upon the addition of H_2_O and Fe^3+^ aqueous solutions with the same volume ([Fe^3+^] = 10^-3^ M).


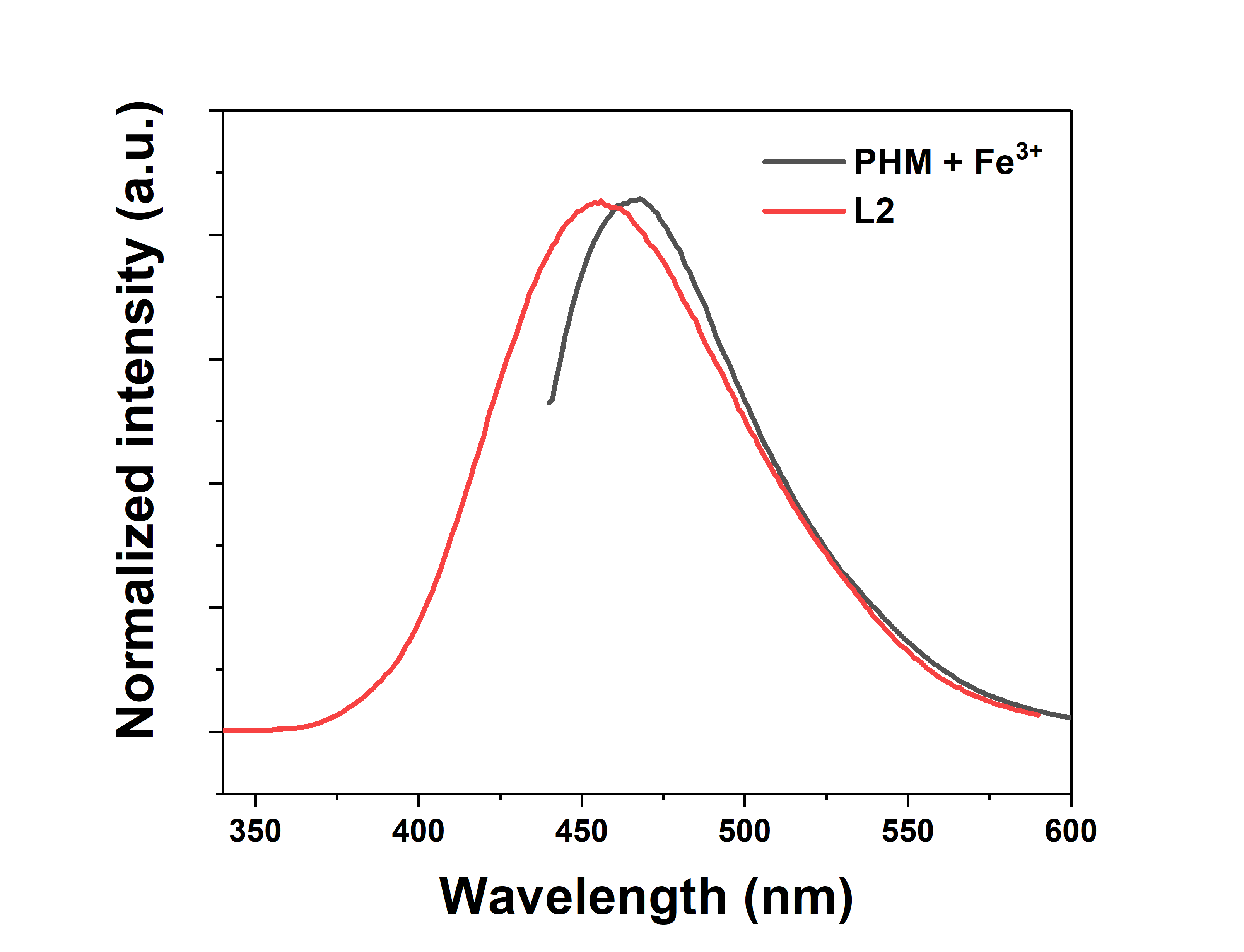


**Figure S24.** Normalized fluorescence spectra of PHM after Fe^3+^ addition (black) and L2 (red).


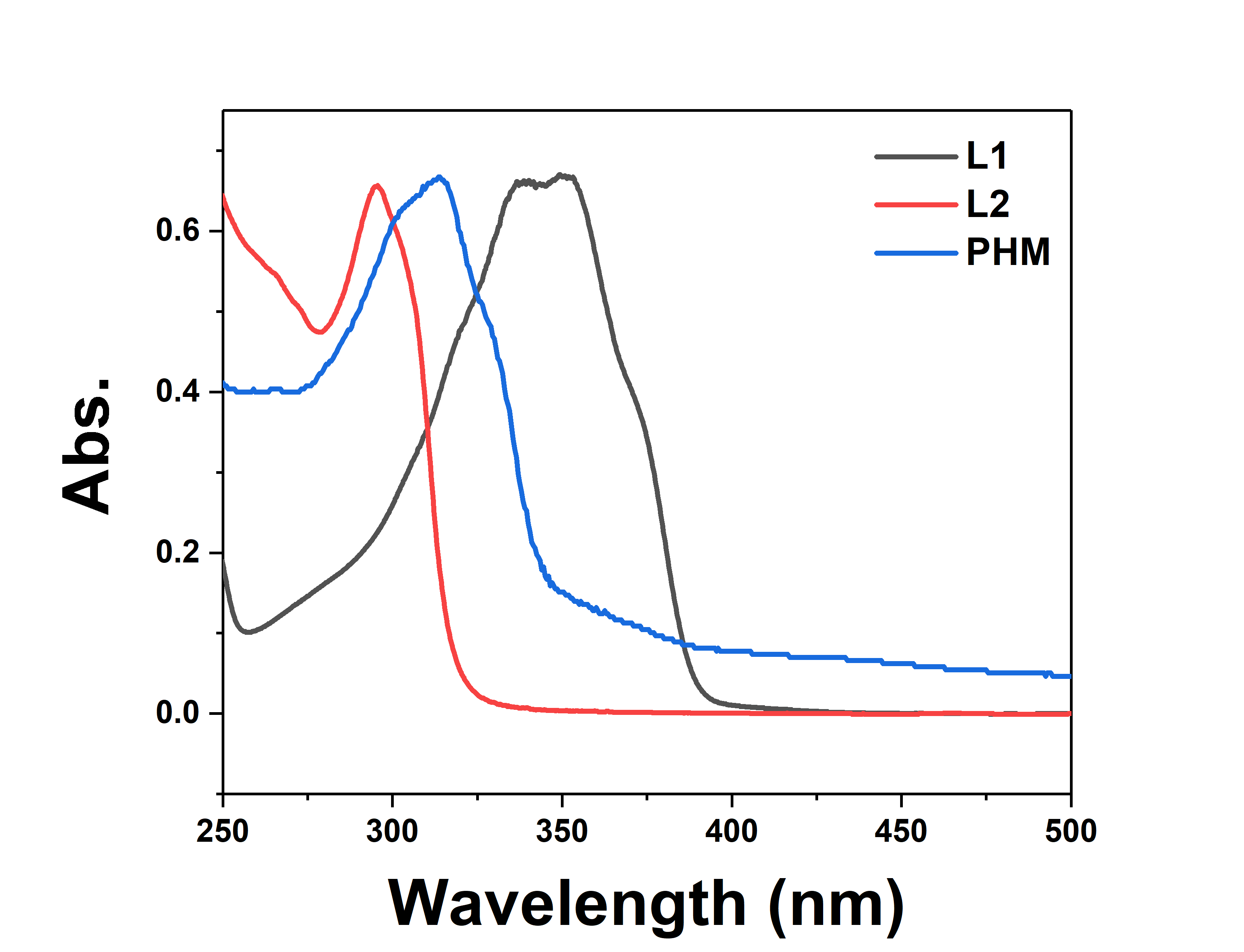


**Figure S25.** UV-vis absorption spectra of L1 (black), L2 (red), and PHM (blue).


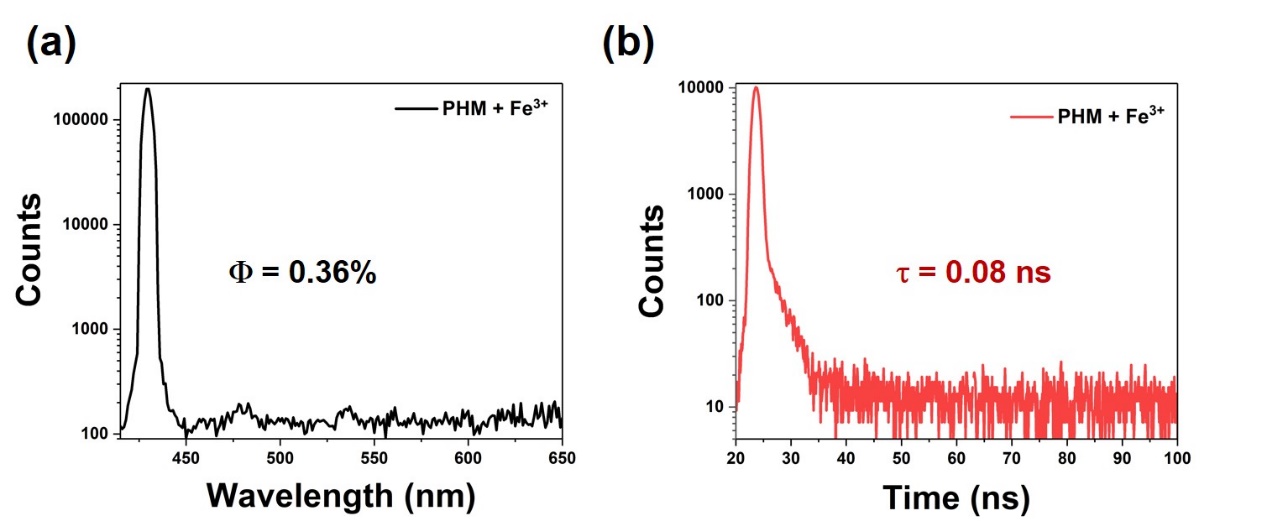


**Figure S26.** (a) Quantum yield and (b) fluorescence lifetime of PHM after treatment of Fe^3+^.


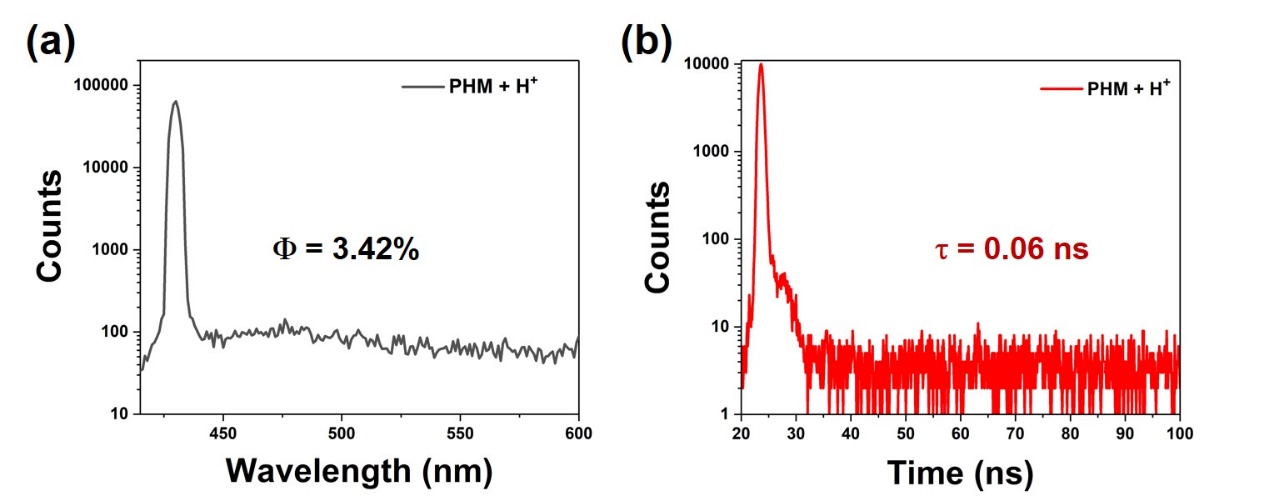


**Figure S27.** (a) Quantum yield and (b) fluorescence lifetime of PHM after treatment of H^+^.


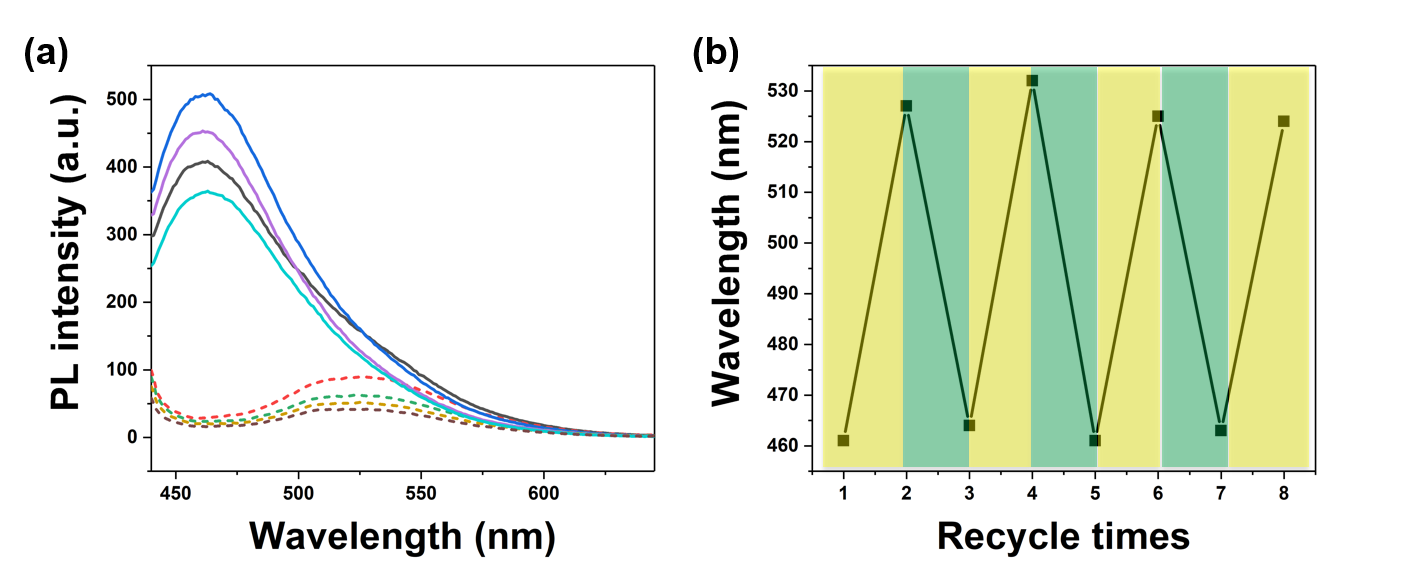


**Figure S28.** (a) Fluorescence spectra of PHM upon alternate treatment of HCl (solid lines) and NaOH (dashed lines); (b) Acid-base recycles of PHM emission wavelengths.


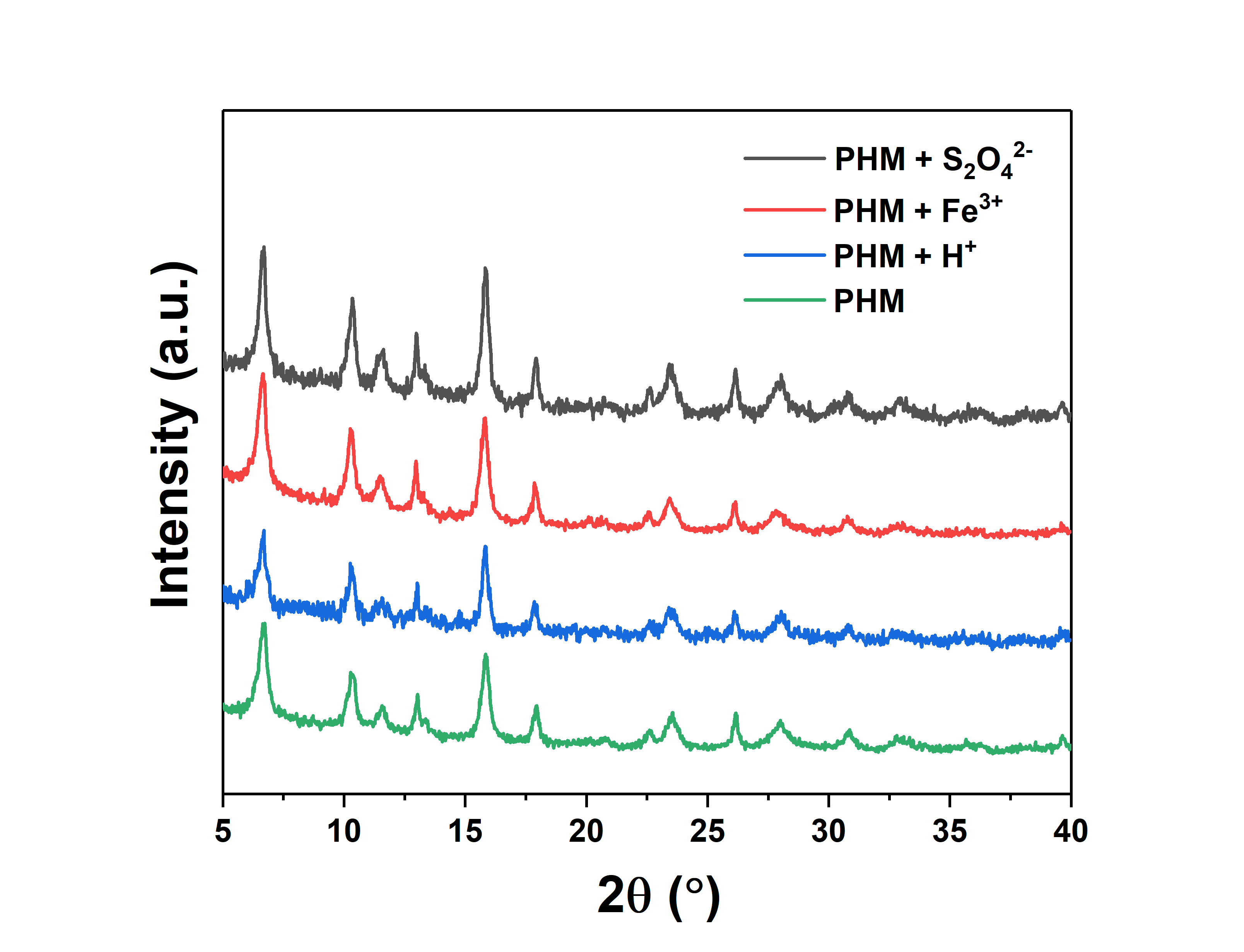


**Figure S29.** PXRD patterns of PHM (green), PHM treated with H^+^ (blue), PHM treated with Fe^3+^ (red), and PHM treated with Fe^3+^ (black), whereby the PHM powder was soaked in solutions containing excessive amounts of Fe^3+^ and H^+^, and then dried thoroughly before tested.

**Table S5.** Fluorescence parameters of PHM under different circumstances (solid state, suspensions in water, DMSO, Fe^3+^, H^+^, and S_2_O_4_^2-^

|  | λ_abs_ (nm) | λ_em_ (nm) | Φ (%) | τ (ns) | K_r_ (s^-1^) | K_nr_ (s^-1^) |
| --- | --- | --- | --- | --- | --- | --- |
| PHM  PHM in H_2_O  PHM in DMSO | 386  310  358 | 551  530  420 | 14.61  28.05  39.51 | 6.35  7.11  0.17 | 2.30×10^7^  3.94×10^7^  2.32×10^9^ | 1.34×10^8^  1.01×10^8^  3.56×10^9^ |
| PHM + Fe^3+^ | 245, 364 | 468 | 0.36 | 0.08 | 4.50×10^7^ | 1.25×10^10^ |
| PHM + H^+^ | 245, 364 | 471 | 3.42 | 0.06 | 4.28×10^8^ | 1.61×10^10^ |
| PHM + S_2_O_4_^2-^ | 316 | 506 | 2.76 | 0.54 | 5.11×10^7^ | 1.80×10^9^ |

## 7. References

S1 Olesinska M, Wu G, Gomez-Coca S, Anton-Garcia D, Szabo I, Rosta E*, et al.* Modular supramolecular dimerization of optically tunable extended aryl viologens. *Chem Sci* 2019,**10**:8806-8811.

S2 Liu P, Li Z, Shi B, Liu J, Zhu H, Huang H. Formation of Linear Side-Chain Polypseudorotaxane with Supramolecular Polymer Backbone through Neutral Halogen Bonds and Pillar[5]arene-Based Host-Guest Interactions. *Chem Eur J* 2018,**24**:4264-4267.

S3 Wu H, Chen Y, Zhang L, Anamimoghadam O, Shen D, Liu Z*, et al.* A Dynamic Tetracationic Macrocycle Exhibiting Photoswitchable Molecular Encapsulation. *J Am Chem Soc* 2019,**141**:1280-1289.
